# Size-isolation of superparamagnetic iron oxide nanoparticles improves MRI, MPI and hyperthermia performance

**DOI:** 10.1186/s12951-020-0580-1

**Published:** 2020-01-28

**Authors:** Seyed Mohammadali Dadfar, Denise Camozzi, Milita Darguzyte, Karolin Roemhild, Paola Varvarà, Josbert Metselaar, Srinivas Banala, Marcel Straub, Nihan Güvener, Ulrich Engelmann, Ioana Slabu, Miriam Buhl, Jan van Leusen, Paul Kögerler, Benita Hermanns-Sachweh, Volkmar Schulz, Fabian Kiessling, Twan Lammers

**Affiliations:** 1 Institute for Experimental Molecular Imaging, Faculty of Medicine, RWTH Aachen University, Forckenbeckstr. 55, 52074 Aachen, Germany; 2 Department of Biomolecular Sciences, University of Urbino ‘Carlo Bo’, Via Aurelio Saffi 2, 61029 Urbino, Italy; 3 Department of Scienze E Tecnologie Biologiche, Chimiche E Farmaceutiche (STEBICEF), University of Palermo, Palermo, Italy; 4 Institute of Applied Medical Engineering, Helmholtz Institute, Faculty of Medicine, RWTH Aachen University, Pauwelsstrasse 20, 52074 Aachen, Germany; 5 Electron Microscopy, Institute of Pathology, Faculty of Medicine, RWTH Aachen University, Pauwelstrasse 30, 52074 Aachen, Germany; 6 Institute of Inorganic Chemistry, RWTH Aachen University, Landoltweg 1, 52056 Aachen, Germany; 7 Department of Pharmaceutics, Utrecht University, Utrecht, The Netherlands; 8 Department of Targeted Therapeutics, University of Twente, Enschede, The Netherlands

**Keywords:** Iron oxide nanoparticles, SPION, MRI, MPI, Hyperthermia

## Abstract

Superparamagnetic iron oxide nanoparticles (SPION) are extensively used for magnetic resonance imaging (MRI) and magnetic particle imaging (MPI), as well as for magnetic fluid hyperthermia (MFH). We here describe a sequential centrifugation protocol to obtain SPION with well-defined sizes from a polydisperse SPION starting formulation, synthesized using the routinely employed co-precipitation technique. Transmission electron microscopy, dynamic light scattering and nanoparticle tracking analyses show that the SPION fractions obtained upon size-isolation are well-defined and almost monodisperse. MRI, MPI and MFH analyses demonstrate improved imaging and hyperthermia performance for size-isolated SPION as compared to the polydisperse starting mixture, as well as to commercial and clinically used iron oxide nanoparticle formulations, such as Resovist® and Sinerem®. The size-isolation protocol presented here may help to identify SPION with optimal properties for diagnostic, therapeutic and theranostic applications.
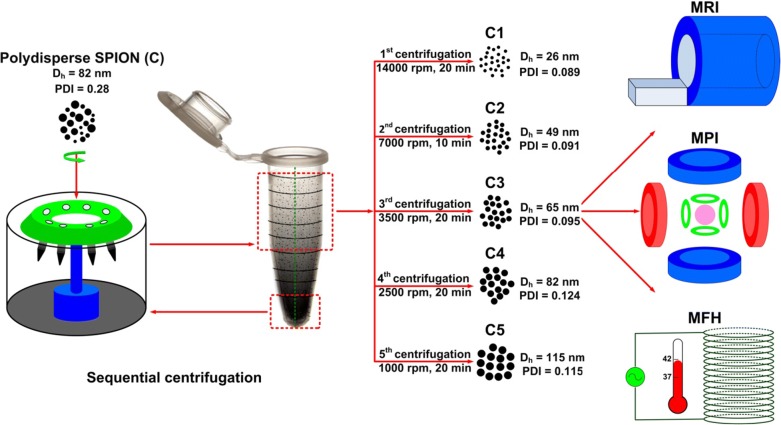

## Introduction

Superparamagnetic iron oxide nanoparticles (SPION) are widely used for biomedical applications, including magnetic resonance imaging (MRI), magnetic particle imaging (MPI), magnetic fluid hyperthermia (MFH), separation of biomolecules, and targeted drug and gene delivery [1–3]. This widespread list of applications not only results from the magnetic properties of SPION, but also from the capability of synthesizing them in different sizes and shapes. For all of the above applications, SPION should ideally have a high magnetization value, a size below 100 nm and a narrow size distribution [4, 5].

SPION are typically based on Fe_3_O_4_ and/or Fe_2_O_3_. They can be synthesized using various methods, such as co-precipitation [5, 6], thermal decomposition [7], sol–gel [8], microemulsion [9], hydrothermal [10], and electrochemical synthesis [11]. The co-precipitation technique is among the most successful, most commonly employed and most cost-effective methods for high-yield synthesis. However, strategies are needed to overcome the most important limitation of this method, i.e. the very broad particle size distribution of the resulting SPION mixture [5, 6].

In this study, we describe a straightforward, easily implementable and broadly applicable centrifugation protocol to obtain relatively monodisperse SPION from a polydisperse starting mixture prepared using the co-precipitation technique. As a result of their refined size distribution, the obtained optimized SPION dispersions showed substantially improved performance in MRI, MPI and MFH compared to the crude starting formulation, as well as to commercial SPION products, such as Resovist® and Sinerem®.

In this context, it is important to keep in mind that not the centrifugation protocol per se, but the eventual development of a SPION formulation with a very well-defined size and with a very narrow size distribution (and its consequent more optimal use for diagnostic and therapeutic purposes) is the objective of our work. Thus far, no systematic study has been published on SPION size-isolation via sequential centrifugation, and no systematic analysis is available in which the performance of five size-isolated SPION sub-fractions (and clinically/commercially relevant controls) is head-to-head compared in MRI, MPI and MFH setups.

## Results and discussion

### SPION preparation and size-isolation

**Fig. 1:**
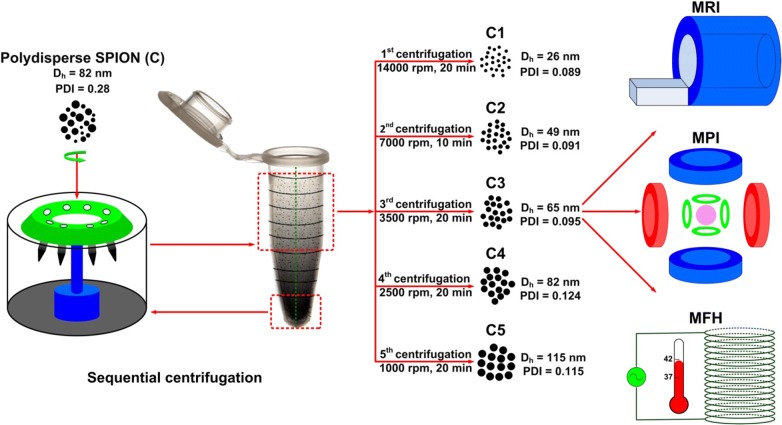
SPION size-isolation via sequential centrifugation. Schematic overview of the centrifugation protocol to obtain monodispersed SPION with different hydrodynamic diameters from a crude mixture of polydisperse SPION. The polydisperse SPION sample (C) was transferred into 1.5 ml Eppendorf tubes and centrifuged at 14,000 rpm for 20 min. The resulting 1 ml of supernatant was collected (C1). 0.1 ml of the bottom compartment in the Eppendorf tube was resuspended in water and again centrifuged, and the top 1 ml was collected (C2). These steps were repeated multiple times, with optimized centrifugation times and speeds, to obtain three additional fractions of monodisperse SPION samples (C3–C5). The different fractions were subsequently analyzed for magnetic resonance imaging (MRI), magnetic particle imaging (MPI) and magnetic fluid hyperthermia (MFH) performance, and compared to the crude sample (C), to Resovist® and to Sinerem®

Prototypic citrate-coated SPION were prepared via the standard co-precipitation technique, under nitrogen atmosphere [5, 6] (see “Experimental” section for details). Based on this highly polydisperse starting batch, which we refer to as the “crude sample”, five sequential rounds of centrifugation were performed to obtain much more monodispersed SPION subfractions. To this end, as depicted schematically in Fig. 1, the crude sample was transferred into 1.5 ml Eppendorf tubes and centrifuged at 14,000 rpm for 20 min. The resulting 1 ml of supernatant was collected and referred to as the “C1 sample”. Subsequently, 0.1 ml of the bottom compartment in the Eppendorf tube that contained the largest nanoparticle fraction was resuspended in water. The obtained dispersion was then again centrifuged, the top 1 ml was collected as the “C2 sample”, and the bottom 0.1 ml was again resuspended and re-centrifuged. These steps were sequentially repeated to obtain five fractions of relatively monodisperse SPION samples. These fractions are referred to as C1–C5. The crude starting mixture, Resovist® and Sinerem® are referred to as C, R and S, respectively. Multiple systematic experiments were performed to identify the optimal centrifugation speeds and times for obtaining monodispersed SPION with well-defined sizes. The optimum conditions for size-isolation are presented in Fig. 1. The production efficiencies of the size-isolated fractions C1, C2, C3, C4 and C5 were approximately 7, 29, 23, 18 and 11%, respectively.

Despite the large number of previous publications describing the synthesis of iron oxide nanoparticles, the tools and technologies for their size separation are relatively limited. Techniques employed to control average particle size and polydispersity can be based on the use of magnetic/electric fields, porous media, and mass- and density-based purification [12–14]. Fortin and colleagues for instance synthesized citrate-coated nanocrystals of maghemite and cobalt ferrite by alkaline co-precipitation, and size-sorted the nanoparticles by successive electrostatic phase separation [15]. Magnetic field-flow fractionation (MFFF) uses a homogeneous external magnetic field applied orthogonal to the direction of flow, to achieve efficient separation of particles [12]. Nonmagnetic size-exclusion chromatography (SEC) is another frequently used method for size separation of iron oxide nanoparticles. The fractions separated by SEC and MFFF have similar size distributions. However, MFFF is faster and has a higher capacity [12, 16]. In addition to the above techniques, differential magnetic catch-and-release (DMCR) has recently been established to size-sort magnetic nanoparticles. DMCR, like MFFF, relies on an external magnetic field to separate magnetic species [17]. High-gradient magnetic separation (HGMS) is a column flow method used to isolate iron oxide nanoparticles from a nonmagnetic medium [18]. Capillary electrophoresis (CE) is used for the separation of colloidal nanoparticles in an electric field. CE requires specialized equipment, because of the high electric field. Electrical field-flow fractionation (ElFFF) separates iron oxide nanoparticles based on their size and electrophoretic mobility but without the drawbacks of CE [12, 16]. As compared to the above techniques, the here presented centrifugation method is somewhat more time- and labor-intensive, but it is also easier to perform and more broadly applicable, because it does not require specialized equipment.

### Particle size, size distribution and surface charge

**Fig. 2:**
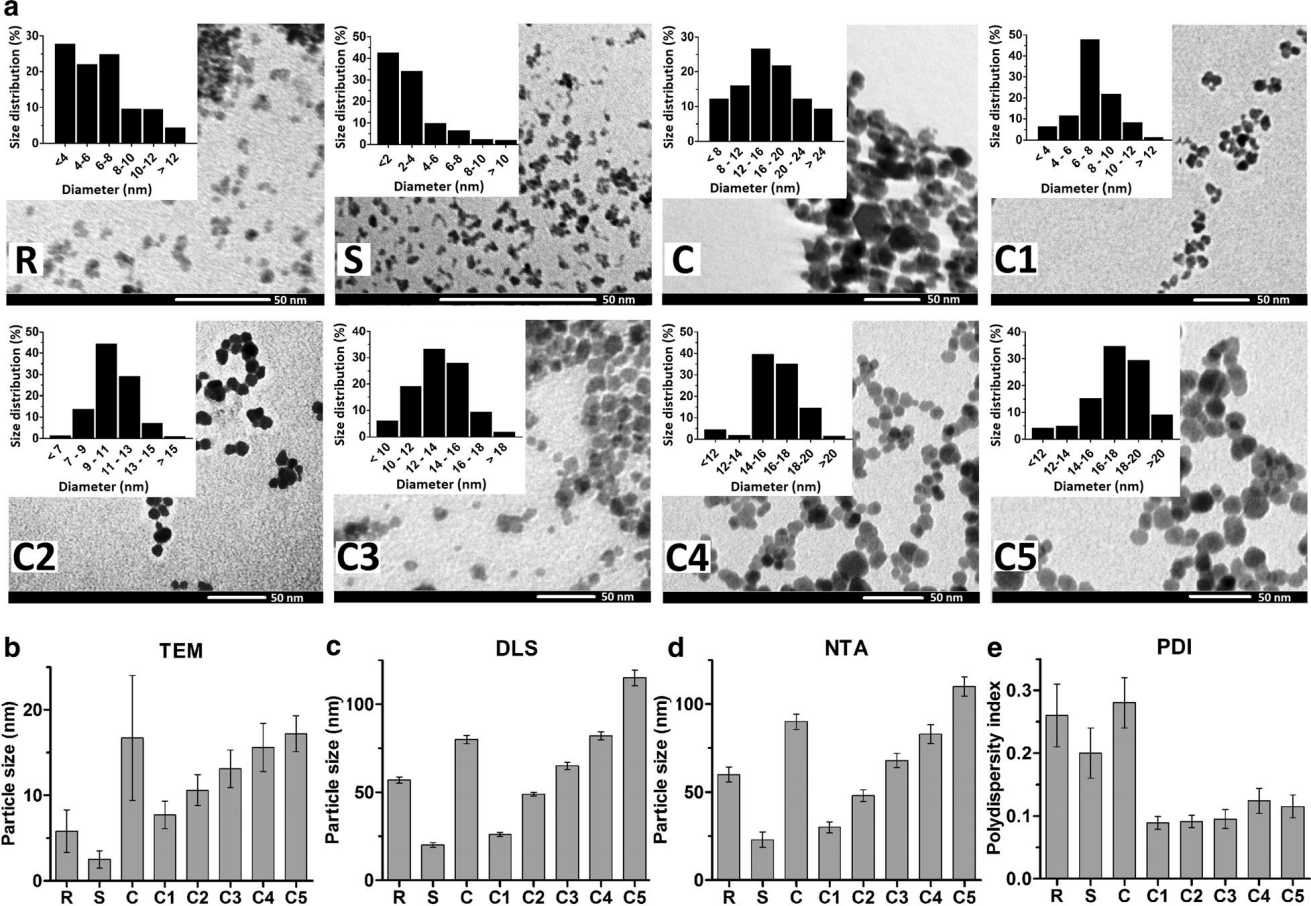
Effect of sequential size-isolation on SPION size and size distribution. **a** TEM images and size distributions obtained by TEM. **b**–**d** Analysis of nanoparticle size obtained using TEM, DLS and NTA. **e** Polydispersity indices (PDI) assessed using DLS for the crude (C), C1–C5, Resovist® (R) and Sinerem® (S) samples. Results represent average ± standard deviation

Figure 2 shows the results obtained using TEM, DLS and NTA on the size and size distribution of the SPION formulations prepared and evaluated in this study. The reported TEM values which correspond to the average size were calculated on the basis of manually measuring at least 100 randomly chosen particles, using Image SP Viewer software. The average core sizes of the samples C1, C2, C3, C4 and C5 were 7.7 ± 1.6, 10.6 ± 1.8, 13.1 ± 2.2, 15.6 ± 2.8 and 17.2 ± 2.1 nm, respectively (Fig. 2a, b). This indicates that all five fractions are superparamagnetic, as SPION typically present superparamagnetic behavior when their core size is below 20 nm [5]. The corresponding average hydrodynamic diameters obtained by DLS-based on intensity—for the five samples were 26.3 ± 1.2, 49.4 ± 1.1, 64.8 ± 2.1, 82.1 ± 2.3 and 114.6 ± 4.4 nm (Fig. 2c). The average sizes obtained using NTA were comparable to the values observed in DLS (Fig. 2d). The numerical values corresponding to the results presented in Fig. 2b–d are provided in Additional file 1: Table S1. The fact that the TEM sizes are smaller than those obtained via DLS and NTA can be explained by keeping in mind that DLS and NTA measure the hydrodynamic diameter of the citrate-coated SPION in aqueous solution incorporating surface-bound water layers in their measurement, while TEM determines the actual core size of dried nanoparticle formulations.

The results obtained using DLS, NTA and TEM demonstrate that both the core size and the hydrodynamic diameter gradually increase upon employing our centrifugation protocol. In this regard, it is important to note that from C1 to C5, the increase in hydrodynamic diameter (DLS) is much larger than the increase in core size (TEM). Equally important is the notion that the polydispersity indices (PDI) obtained from DLS confirmed that the samples C1–C5 have a much narrower size distribution than the crude sample, and also than Resovist® and Sinerem®. The PDI for the crude sample, for Resovist® and for Sinerem® were 0.28 ± 0.04, 0.26 ± 0.05 and 0.20 ± 0.04, respectively, while for C1–C5, all PDI’s were approximately 0.10 (Fig. 2e). The size distribution results obtained by TEM are in good agreement with this (see the insets in Fig. 2a and the data presented in Fig. 2e). Based on these results, it is concluded that our sequential centrifugation protocol is highly useful for achieving relatively monodisperse SPION formulations. Consequently, it is considered to be a useful alternative to more complex synthetic methods to obtain relatively uniform SPION, such as thermal decomposition, which requires very high temperatures and which critically depends on efficient and tailored means for surface modification to eventually obtain water-dispersible SPION formulations [7].

We also determined the zeta potential for the differently sized iron oxide nanoparticle samples (Additional file 1: Figure S1). The results confirm the expected highly negatively surface charge for all size-isolated fractions (C1–C5), which contributes to their high colloidal stability.

### SPION biocompatibility

Almost all SPION formulations were found to be biocompatible. Additional file 1: Figures S2–S4 document the observed cytotoxicity for the crude, C1–C5, Resovist® and Sinerem® samples studied by XTT, LDH and ROS assays. XTT analysis at iron concentrations of 0.1 and 1.0 mM showed no significant differences in the viability of NIH3T3 cells upon incubation with the samples C1–C5 as compared to Resovist® and Sinerem®. Interestingly, at iron concentrations of 5 and 10 mM, XTT-based viability assessment indicated that all monodispersed samples except for C1 had an even higher biocompatibility than Resovist® and Sinerem® (Additional file 1: Figure S2). The XTT findings were confirmed using the LDH assay (Additional file 1: Figure S3). At iron concentrations of 0.1 and 1 mM, no changes in NIH3T3 membrane damage were noted for C1–C5 as compared to Resovist® and Sinerem®, while at iron concentrations of 5 and 10 mM, LDH values (and membrane damage) were lower than for Resovist® and Sinerem® (again except for the smallest-sized batch C1). In line with this, analysis of ROS production in NIH3T3 cells showed that there was no significant change in the ROS content of cells exposed to the monodispersed samples C1–C5 as compared to the crude sample, Resovist® and Sinerem® (Additional file 1: Figure S4). Together, these results demonstrate that all monodispersed samples except for C1 have negligible toxicity. The higher cytotoxicity associated with the smallest particles is assumed to result from a more rapid and more extensive cellular uptake, as well as from a relatively larger surface area [19–21].

### SPION stability in physiological media

All size-isolated SPION samples showed excellent stability in DI water (see columns 4 and 5 of Additional file 1: Table S1; demonstrating stable dispersion up to 6 months). This can be attributed to the highly negatively charged surface of the SPION. All SPION formulations also showed high colloidal stability in physiological media, i.e. in fetal bovine serum (FBS) and in bovine serum albumin (BSA). The monitoring of the samples by visual inspection up to 24 h implied the absence of aggregation of SPION (see Additional file 1: Figures S5a and S6a). In line with this, the hydrodynamic diameters and PDI obtained using DLS for 2, 6 and 24 h of incubation in physiological media did not show significant changes in size and size distribution (see Additional file 1: Figures S5b, c, S6b, c and Table S1). In good agreement with our findings, Yu et al. synthesized two different types of SPION with different surface coatings: tetramethylammonium hydroxide-coated SPION (T-SPION) and citrate-coated SPION (C-SPION). The C-SPION showed robust stability in biological media, while T-SPION aggregated rapidly in all media evaluated [22].

### Magnetic properties

**Fig. 3:**
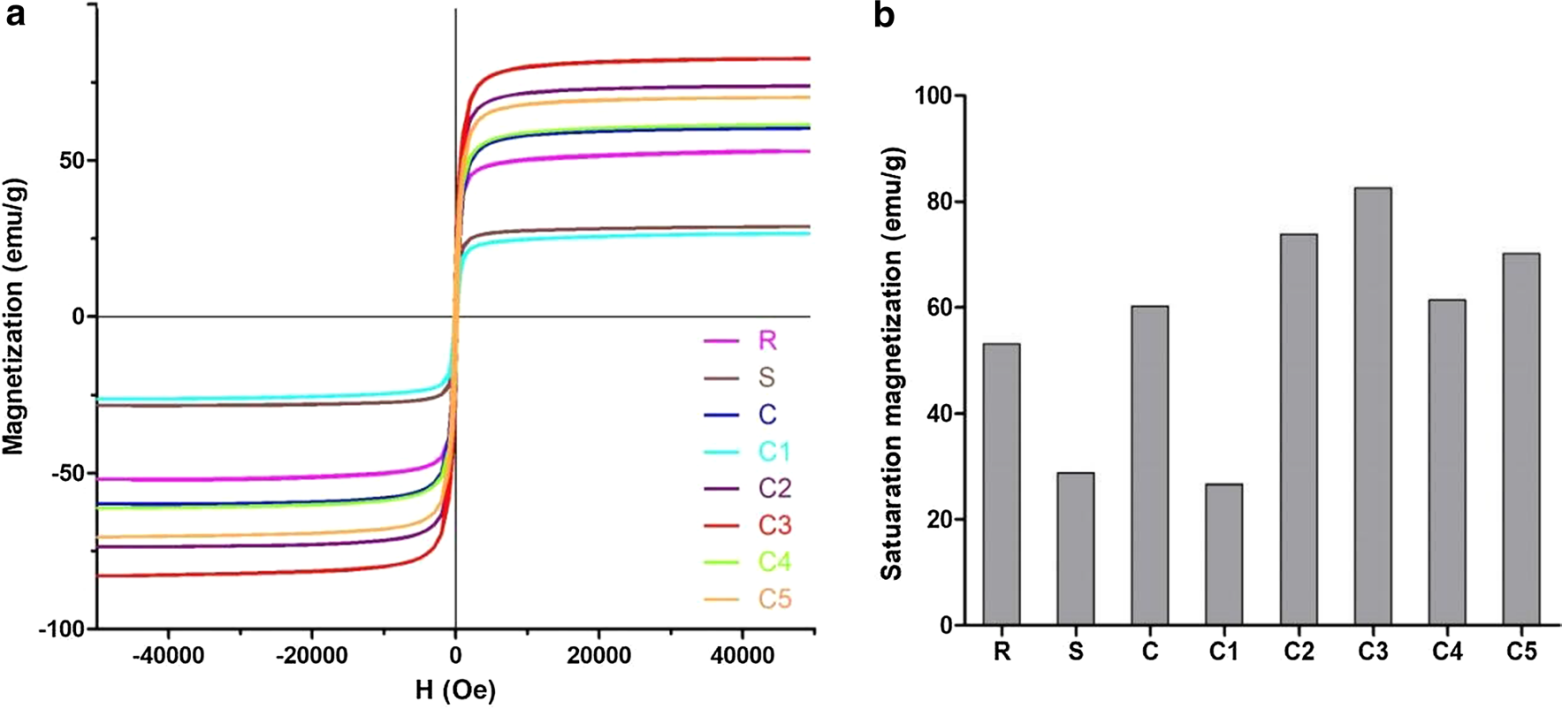
Magnetic characterization. **a** Field-dependent magnetization at 5 K. **b** Saturation magnetization at 5 K of the crude SPION mixture (C), the size-isolated samples C1–C5, Resovist® (R) and Sinerem® (S). Results were normalized to Fe content

Field-dependent magnetization analysis of the C1–C5 samples showed no discernible hysteresis, demonstrating that they are superparamagnetic (Fig. 3a). For biomedical applications, iron oxide nanoparticles with superparamagnetic behavior are preferred, because in case of superparamagnetic materials, the magnetization drops to zero after removing the applied magnetic field. This implies that due to lack of coercive forces or remanence, it keeps the nanoparticles from sticking together, avoiding aggregation and the formation of clots in the blood stream, which could lead to serious adverse events [23].

The saturation magnetizations (M_s_) of samples were very high, indicating an excellent magnetic response to the magnetic field (Fig. 3b). Three important observations were obtained by these analyses: first, comparing the M_s_ values of the samples C2 and C3 at 5 K and 30 kOe (73.8 and 82.5 emu/g, respectively) to those of Resovist® and Sinerem® (53.1 and 28.8 emu/g, respectively) illustrates the good magnetic properties of C2 and C3. Second, the M_s_ values for C2 and C3 are approximately three-quarters of the M_s_ value of bulk magnetite, which is ~ 100 emu/g at 5 K and 30 kOe [24]. Third, the magnetization reaches 94% of its maximum value for C2 and 93% of its maximum value for C3 in magnetic fields as low as 5 kOe, underlining the suitability of these samples for the envisaged applications. Field-cooled (FC) magnetization measurements were also carried out, in an applied field of 1000 Oe, at temperatures ranging from 5 to 300 K. As shown in Additional file 1: Figure S7, the FC curves demonstrate only a very little decrease with temperature for all nanoparticle samples tested, and the results obtained are in good agreement with those of saturation magnetization analyses.

Both MRI and MPI rely on the use of magnetic nanoparticles with strong saturation magnetization, high magnetic susceptibility and no coercivity. Similarly, also for MFH, the amount of saturation magnetization should be as high as possible, to guarantee efficient heating under an alternating magnetic field [23]. Saturation magnetization of SPION depends not only on core size, but also on other parameters, such as size distribution, type of coating, chemical composition (with magnetite being better than maghemite) and crystalline structure. Generally, a larger particle size results in higher saturation magnetization values and in a better performance in MRI, MPI and MFH. However, when the particle size is too large, magnetic nanoparticles become ferromagnetic and the saturation magnetization drops, which is undesired for biomedical applications. For the C1–C5 samples, field-dependent magnetization analysis revealed that all fractions are in the superparamagnetic range. Increasing the size gradually approaches ferromagnetic behavior, explaining the somewhat lower saturation magnetization values for C4 and C5 as compared to C2 and C3. Also, the low saturation magnetization for C4 and C5 compared to C2 and C3 could be explained on the basis of a more polycrystalline structure of the samples. Conversely, it is important to keep in mind that smaller-sized nanoparticles are typically preferred in vivo, e.g. because they can more readily exploit vascular leakiness in tumors and at sites of inflammation, and because they allow for deeper target tissue penetration. These considerations exemplify that it is crucial to identify the optimal size for the anticipated biomedical application [25, 26], and they underline the importance of developing tools, such as the centrifugation protocol presented here, to prepare SPION formulations with distinct sizes and with low polydispersity.

Another important thing to keep in mind is that sometimes the saturation magnetization is found to be lower than expected. This reduction in magnetic performance of the nanoparticles can be attributed to the existence of a "magnetically dead layer" on their surfaces. Because of this magnetically dead layer, the magnetic diameter is smaller than the physical diameter, sometimes by several nanometers. Saturation magnetization is proportional to the magnetic diameter, not physical diameter [27–29]. As an example to illustrate this, Unni and colleagues synthesized two series of iron oxide nanoparticles with a similar diameter of 21 nm via thermal decomposition; the MS value was 17 emu/g for one nanoparticle, and 74 emu/g for the other [27]. Kemp et al. produced monodisperse magnetite nanoparticles with diameters in the range between 15 and 30 nm by thermolysis and they varied oleic acid ratios for size control. With increasing particle size, there was no clear trend in saturation magnetization (sometimes increasing and sometimes decreasing) [28]. Such irregularities were also observed by Baaziz et al. for iron oxide nanoparticles with diameters between 4 and 28 nm [29]. The lower MS values for the samples C4 and C5 as compared to C2 and C3 can be explained by taking the above notions into account.

### Magnetic resonance imaging

**Fig. 4:**
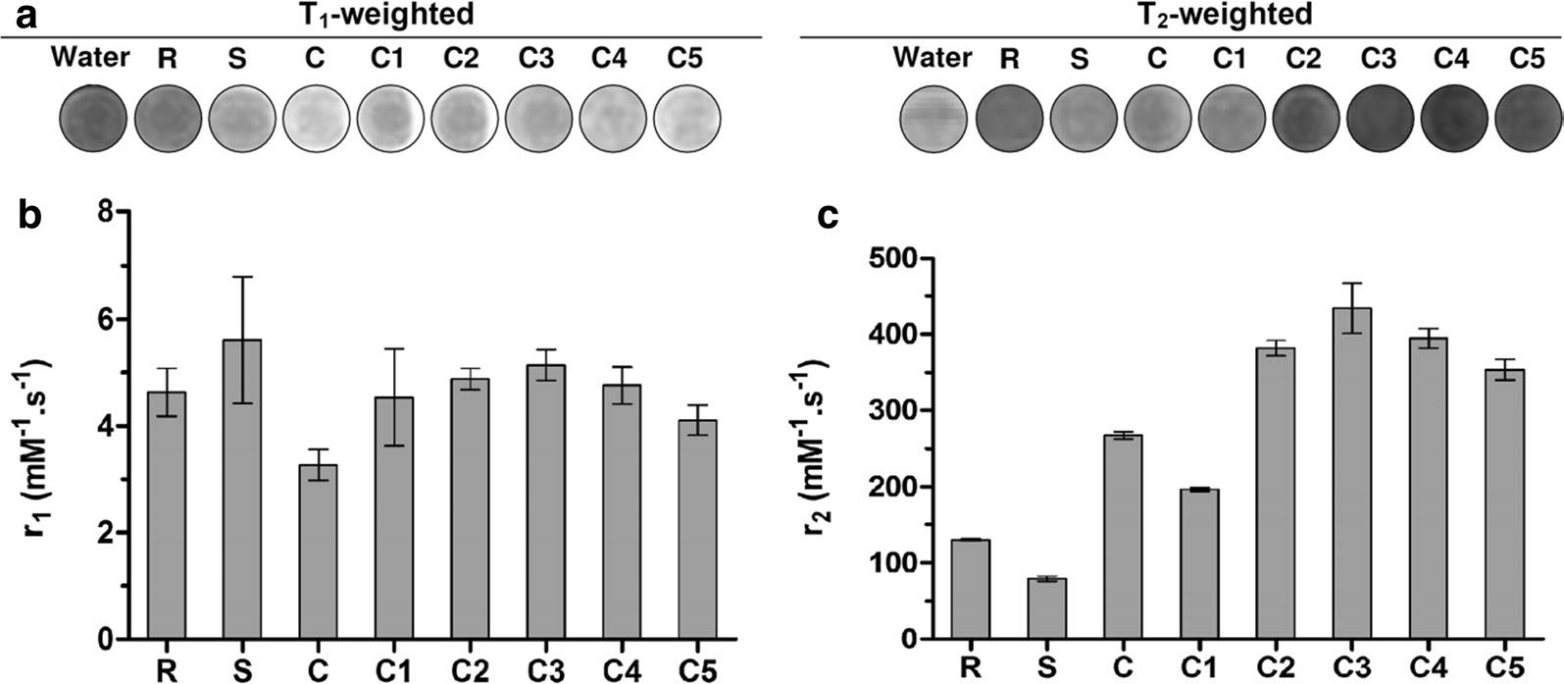
Magnetic resonance imaging of size-isolated SPION. MRI of the crude, C1–C5, Resovist® and Sinerem® samples upon characterization on a 3 T clinical scanner. **a** T_1_- and T_2_-weighted MR images of the samples at a concentration of 0.01 mM. MR images for other SPION concentrations are provided in Additional file 1: Figure S8. **b** and **c** Longitudinal (r_1_) and transversal (r_2_) relaxivities of the samples in water. Values represent average ± standard deviation of three independent samples

All SPION samples showed excellent performance as contrast agent for magnetic resonance imaging (MRI). Figure 4 and Additional file 1: Figures S8–10 show T_1_- and T_2_-weighted MR images and quantification of key MRI parameters for the crude, C1–C5, Resovist® and Sinerem® samples [i.e. relaxivities (r_1_, r_2_), relaxation rates (1/T_1_, 1/T_2_) and relaxivity ratios (r_2_/r_1_)]. Figure 4 indicates that all newly prepared samples, i.e. both the monodisperse and the polydisperse SPION, have transverse relaxivities (r_2_) greater than Resovist® and Sinerem®. Interestingly, while the crude starting mixture and Resovist® were both highly polydisperse, the r_2_ value of the former was found to be two times higher than that of the latter.

After sequential centrifugation, the r_2_ values of the monodisperse SPION gradually increased up until the third round of centrifugation. The C3 sample with 13.1 ± 2.2 nm core size possessed the most optimal MRI capabilities, with an r_2_ value of 434 mM^−1^ s^−1^. It produced 3.3 and 5.5 times more contrast in T_2_-weighted imaging than Resovist® (130 mM^−1^ s^−1^) and Sinerem® (79 mM^−1^ s^−1^), respectively. A number of studies have demonstrated that the core size, the size distribution and the magnetization of SPION are key factors influencing the transverse relaxation rate (1/T_2_) [15, 30]. The trend for the r_1_ values for the samples C1–C5 was found to be similar to that observed for the r_2_ values.

The efficiency of a T2 contrast agent relies on the r2/r1 ratio in addition to the r2 value [31]. In this context, it is important to note that for all size-isolated samples, it can be concluded that there is a specific enhancement of the r_2_/r_1_ ratio in comparison to Resovist® and Sinerem® (Additional file 1: Figure S10), confirming the suitability of these samples for T_2_-weighted MR imaging.

Saraswathy and colleagues synthesized citrate-coated iron oxide nanoparticles with a similar coating and with a similar core size as C3 sample. They employed this SPION formulation for in vivo magnetic resonance imaging of liver fibrosis. The values for r_1_ and r_2_ were 2.69 and 102 mM^−1^ s^−1^, respectively [32]. Comparing the r_2_/r_1_ value of their formulation (i.e. 37.9) to that of our C3 sample (i.e. 84.4) exemplifies the usefulness and the potential added value of our sequential size-isolation protocol. Smolensky et al. investigated the effect of multiple parameters, including particle size and shape, temperature and magnetic field strength, on the longitudinal and transverse relaxivities of iron oxide nanoparticles. According to their findings, r_2_ values increased linearly with increasing core size (from 4.9 to 18 nm), while r_1_ values remained relatively constant for particles with core sizes larger than 8 nm [33]. Surface coating and nanoparticle aggregation are also highly important parameters. Blanco-Andujar and coworkers studied the evolution of r_2_ with SPION aggregate size [34]. In case of small clusters, nanoparticles are homogeneously dispersed in water and protons can readily diffuse between the magnetic cores. Under these conditions, r_2_ values gradually increase with hydrodynamic diameter (up to approx. 80 nm). At a size of 80–90 nm, there is no further increase in r_2_. If the size exceeds 90 nm, r_2_ values start to decrease with increasing size, due to reductions in surface accessibility and proton exchange rate. This trend is in line with our results, showing reductions in r_2_ values when the hydrodynamic diameter goes beyond 70 nm (r_2_ values for C4 and C5 are 398 and 350 mM^−1^ s^−1^, respectively, as compared to 434 mM^−1^ s^−1^ for C3).

### Magnetic particle imaging

SPION are important tracer materials for magnetic particle imaging (MPI). MPI is a novel and increasingly popular hot-spot imaging technique that can be employed to visualize magnetic nanoparticles with very high temporal and spatial resolution. MPI is able to provide real-time 3D imaging information on the localization and concentration of magnetic nanoparticles, and it can be employed for multiple medical imaging applications [35]. The potential usefulness of MPI strongly depends on the availability of size-optimized SPION to generate high quality images. As a matter of fact, MPI contrast generation critically depends on both SPION size and size distribution, since both parameters strongly affect the magnetization response.

Resovist® was originally developed as a contrast agent for MRI. In recent years, it has also been extensively employed for MPI, because of its large magnetic moment. At the moment, Resovist® is the most extensively employed SPION formulation for MPI. From TEM images, it is known that Resovist® mainly consists of particles with an average core diameter of 5.8 ± 2.5 nm, many of which are agglomerated in clusters (Fig. 2a). It is assumed that these aggregates, which are formed by small elementary particles, are responsible for its good MPI performance [26]. However, the MPI performance of Resovist® still leaves significant room for improvement. As result of this, in recent years, ever more scientists have started to work on the development of better SPION formulations for MPI [26, 36].

**Fig. 5:**
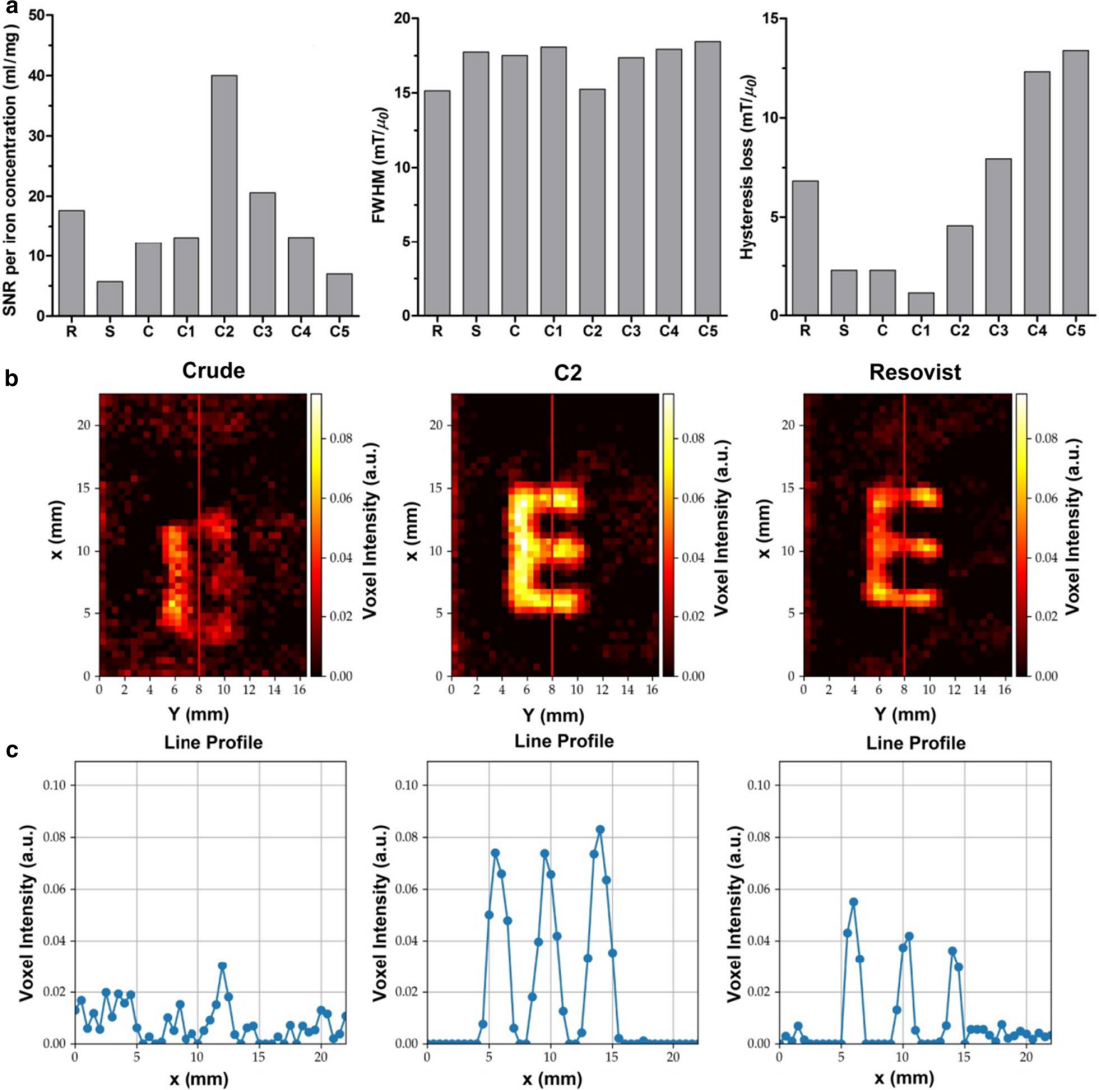
Magnetic particle imaging of size-isolated SPION. **a** Key MPI parameters including normalized signal-to-noise ratios (SNR) of the samples at the 4th harmonic of the MPI drive field as well as full width at half maximum (FWHM) measurements and hysteresis loss analyses of the samples were obtained using magnetic particle spectroscopy (MPS; which is comparable to a zero-dimensional MPI acquisition without the superimposed gradient field measurements). **b** MPI images reconstructed based on “E” shaped phantoms filled with the crude sample, C2 and Resovist®. **c** The intensity line profiles of the red marked lines through the phantoms in **b** are shown. The line profiles show the voxel intensity along the marked line and demonstrate a doubling of signal intensity for C2 in comparison to Resovist®

Figure 5a shows the MPI signal-to-noise (SNR) values of the different SPION formulations used in this study, obtained at the 4th harmonic frequency of the drive field. It also shows the full width at half maximum (FWHM) values, and the hysteresis loss determined from the point spread function (PSF) measurements. To allow for a quantitative comparison, it is generally considered to be sufficient to read the SNR at one harmonic frequency. This is typically the 4th harmonic frequency (Fig. 5a). Additional file 1: Figure S11 shows the SNR values for other harmonic frequencies. To compare the MPI performance of the different samples, SNR values were normalized to the iron concentration inside the probe volume. The normalized SNR values for C2 and C3 were found to be much higher than for all other samples. At the 4th harmonic frequency, the normalized SNR for C2 was 2.3 and 7.0 times higher than for Resovist® and Sinerem®, respectively. In addition, FWHM and hysteresis loss analysis showed that C2 and C3 were almost as good as Resovist®. Lower FWHM and hysteresis loss values refer to a higher achievable spatial resolution and to a lower spatial displacement in MPI, respectively.

To exemplify the MPI imaging capabilities of our size-isolated SPION, we fabricated two phantoms. One was an E-shaped phantom (Fig. 5b), serving as a somewhat more complex structure, made up of single tracer-filled dots of 0.5 mm. The other phantom was V-shaped (Additional file 1: Figure S12a), and consisted of single dots with a diameter of 0.5 mm with an increasing distance between them (2, 3, 4, 5 and 6 mm). Both phantoms were filled with the crude starting mixture, with the C2 sample and with Resovist®, making sure that the iron concentrations were identical. Figure 5c and Additional file 1: Figure S12b show the line profiles of the voxel intensities along the red marked lines for the E and V phantoms, respectively. It can be seen that the lowest and the highest intensities are obtained with the crude and the C2 sample, respectively. The C2 sample produced signal intensities more than two times higher than those of Resovist®. From the MPI parameter analysis as well as from the MPI phantom experiments it can, therefore, be concluded that the C2 (and to a lesser extent also the C3) formulation is a useful alternative for Resovist® and suitable contrast agent for MPI.

### Magnetic fluid hyperthermia

Hyperthermia is a treatment modality in which cancerous tissue is exposed to a supernormal temperature. Cancer cells die as soon as temperatures exceed 42 °C, while normal cells can survive under these conditions [37]. Hyperthermia can be generated using radiofrequency, ultrasound and microwave energy, as well as using magnetic fluid hyperthermia (MFH). In MFH, increased temperatures are created by applying a sinusoidally alternating magnetic field (AMF). When SPION are exposed to an AMF, heat is generated to release the magnetic energy consumed for the alignment of the magnetization of the magnetic particles in the direction of the applied magnetic field. In principle, three mechanisms are responsible for heat dissipation, which can act separately or simultaneously, depending on the nanoparticle properties: (1) hysteresis power loss, originating from the irreversibility of the magnetization process, (2) Néel relaxation, conditioned by the rotation of the magnetic moments of the particles, and (3) friction losses due to Brownian rotation of the magnetic particles as a whole. As a result of these three mechanisms, SPION and magnetic temperature gradually increase in an AFM until a saturation temperature is achieved [37, 38]. In a cellular environment, however, SPION are immobilized inside lysosomes and form agglomerates [39, 40]. This leads to partial blocking of the above-mentioned Brownian relaxation and to a drop in heating efficiency. In consequence, depending on the mechanism responsible for heat generation for a specific nanoparticle type, the in vivo hyperthermia performance could significantly decrease [30].

**Fig. 6:**
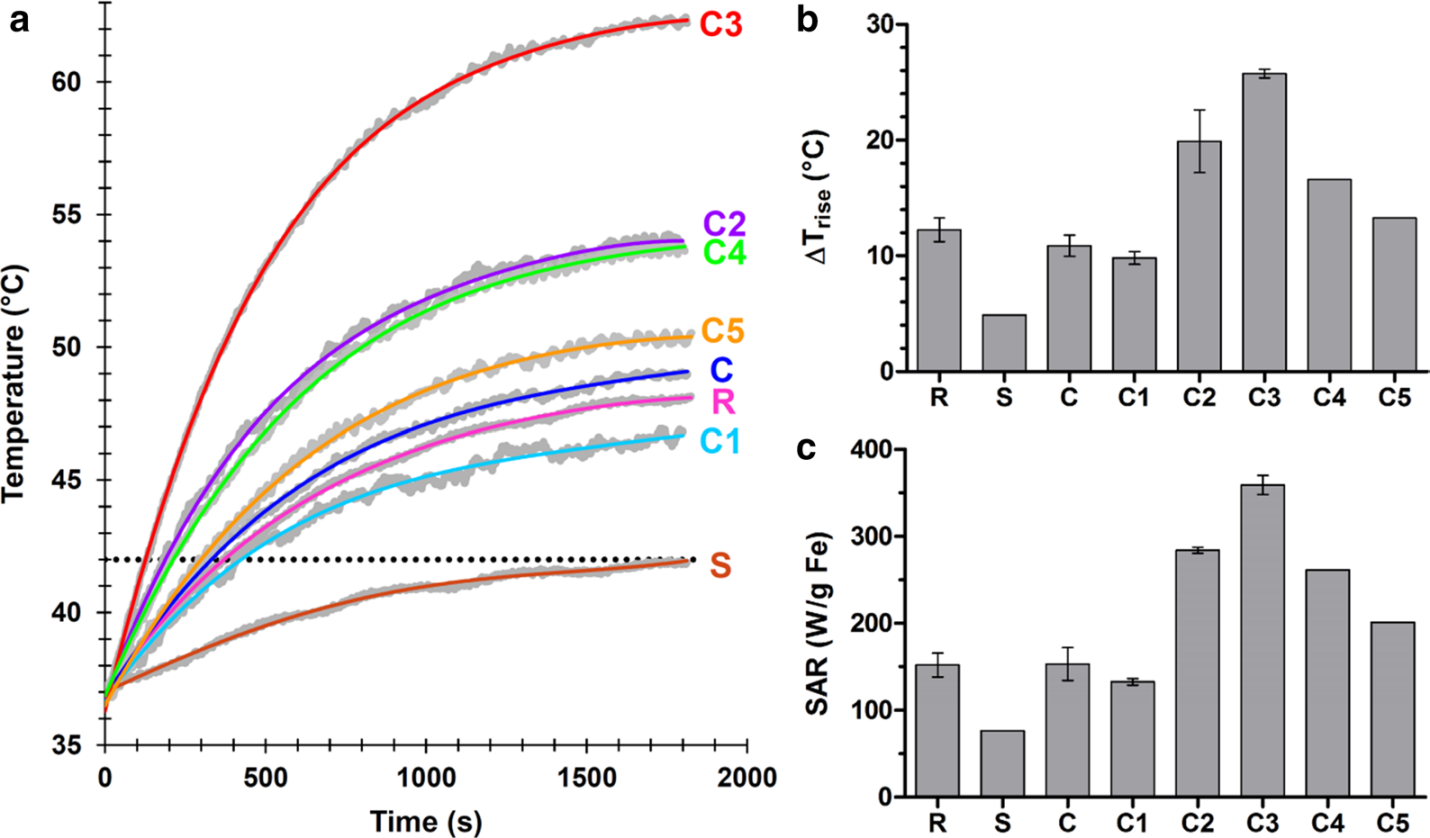
Magnetic fluid hyperthermia using size-isolated SPION. **a** Time–temperature curves obtained upon exposing the crude, C1–C5, Resovist® and Sinerem® samples to an alternating magnetic field (AMF). The frequency and amplitude of the AMF were 186 kHz and 46 kA m^−1^, respectively. The iron concentration was 9 mM for all samples. A Box-Lucas curve was fitted to each data set. **b** Difference between initial and maximum temperatures after 30 min of field exposure (ΔT_rise_). **c** Specific absorption rate values (SAR; calculated on the basis of Additional file 1: Equations S5, S9). Values represent average ± standard deviation of three separate experiments

Figure 6a depicts the time–temperature curves for the monodisperse SPION batches C1-C5, as well as for the crude sample C, Resovist® and Sinerem® in a low-frequency AMF. The iron concentration of all samples was 9 mM and the dispersant media was DI water. For all size-isolated samples except for C1, the required time for increasing the temperature from 37 to 42 °C (t_H_) was lower than for Resovist® and Sinerem®. In this context, a shorter t_H_ time reflects a better heating performance and contributes to shorter AMF application times in hyperthermia-based cancer treatment. The shortest t_H_ value was achieved using C3, having a core size of 13 nm. For this sample, the time to increase the temperature from 37 to 42 °C was 128 s, which was approximately 3 times faster than for Resovist® (t_H_ = 374 s).

In addition to t_H_, the specific absorption rate (SAR) is an important quantitative parameter to determine the suitability of SPION formulations for MFH. From Additional file 1: Equations S7 and S8, it can be deduced that the SAR is directly proportional to ΔT_rise_ which is defined as the difference between the maximum temperature reached during AMF exposure and the initial temperature (in this specific case 37 °C). Comparing the ΔT_rise_ and the SAR values of the different formulations shows that the samples with a higher ΔT_rise_ have a higher SAR and consequently a better MFH performance (Fig. 6b, c). For the C3 sample, the SAR was approximately 2.5 times higher than for Resovist®. This indicates that the magnetic power absorbed per unit mass of the C3 sample in the presence of an AMF is ~ 2.5 times higher than that of Resovist®. This high SAR value is expected to be due to a high saturation magnetization arising from individual magnetic anisotropy. Higher SAR values are beneficial from a clinical point of view, as they allow for lower SPION dosing to achieve similar hyperthermia efficacy.

A wide range of SAR values have been reported in the literature for diverse colloidal SPION formulations. SAR values strongly depended on the mean size and monodispersity of SPION, structural and magnetic properties, and the frequency and amplitude of the magnetic field. In the majority of cases, SAR values in the range between 4 and 100 W/g were achieved for commercially available SPION dispersions [41]. For some customized formulations, higher SAR values have been reported. Bakoglidis and colleagues, for instance, synthesized spherical oleic acid-coated SPION with core sizes between 5 and 18 nm by thermal decomposition, and subjected them to MFH, showing maximal performance for 10 nm, with a SAR of 230 W/g. They used hexane as the dispersion medium to maintain a stable suspension of the nanoparticles [42]. For the size-isolated C3 sample, we observed an SAR of 350 W/g, which exceeds this previously reported value by more than 50%. This notion indicates that upon simple and straightforward size-isolation via sequential centrifugation, SPION formulations with optimal performance for biomedical applications can be readily obtained.

## Conclusion

We here present a centrifugation protocol to obtain SPION with well-defined sizes (hydrodynamic diameter: 26.3 ± 1.2, 49.4 ± 1.1, 64.8 ± 2.1, 82.1 ± 2.3 and 114.6 ± 4.4 nm; and core size: 7.7 ± 1.6, 10.6 ± 1.8, 13.1 ± 2.2, 15.6 ± 2.8 and 17.2 ± 2.1 nm) and with a very narrow size distribution (PDI below 0.1) from a polydisperse starting mixture prepared via the co-precipitation technique. The samples obtained upon the 2nd and 3rd round of centrifugation, which had a core size of 10.6 ± 1.8 and 13.1 ± 2.2 nm, and a hydrodynamic diameter of 49.4 ± 1.1 and 64.8 ± 2.1 nm, were found to be optimal for MRI, MPI and MFH application, with an up to 3.3-, 3.3- and 7-fold improved performance as compared to the crude starting mixture, Resovist® and Sinerem®, respectively. Our results demonstrate that simple and straightforward size-isolation helps to improve the performance for biomedical application.

## Experimental

### SPION synthesis

Eight mmol of ferric chloride was dissolved in DI water and mixed for 5 min under mechanical stirring. Subsequently, 4 mmol of ferrous chloride tetrahydrate was added to the solution and mixed for a further 5 min at room temperature. The pH of the solution was adjusted to 11.0 by adding of 1 M aqueous ammonia solution drop-wisely and it was stirred at 25 °C for 30 min under nitrogen atmospher. The formed black-colored iron oxide particles were decanted using a permanent magnet and washed at least three times with DI water. Afterwards, a specific amount of 0.1 M hydrochloric acid was added to the particles and sonicated for 10 min. Following, the citrate solution was added to the mixture and was stirred at 80 °C for 2 h. The citrate-coated polydisperse particles were separated by the use of a permanent magnet and then resuspended in DI water. Finally, the suspension was passed through a 0.2 µm filter to remove the big particles. Additional synthetic details are provided in Additional file 1.

### SPION characterization

The prepared SPION were subjected to several systematic analyses, to assess their properties and performance. The particle size and the size distribution of the crude sample, of the C1–C5 subfractions, and of Resovist® and Sinerem® were investigated by multiple different sizing techniques, including dynamic light scattering (DLS), nanoparticle tracking analysis (NTA) and transmission electron microscopy (TEM). The zeta potential values of the nanoparticles in aqueous solution were measured using a Zetasizer Nano-ZS (Malvern Instruments, Malvern, UK). The iron concentration of the respective samples was measured using the 1,10-phenanthroline assay [43]. We also evaluated the cytotoxicity of the samples. This was done via 2,3-bis-(2-methoxy-4-nitro-5-sulfophenyl)-2H-tetrazolium-5-carboxanilide (XTT), lactate dehydrogenase (LDH) and reactive oxygen species (ROS) assays at multiple different iron concentrations, ranging from 0.1 to 10 mM. The colloidal stability of all size-isolated samples was investigated in two physiologically relevant media. These were fetal bovine serum (FBS), which is the most widely used serum-supplement for the in vitro cell culture, and bovine serum albumin (BSA). Colloidal stability was analyzed upon incubation in FBS and BSA for 2, 6 and 24 h, via visual inspection and DLS analysis. Measurements of magnetic properties, including field-dependent magnetization, saturation magnetization (M_s_) and field-cooled (FC) magnetization, were performed using a Quantum MPMS-5XL SQUID magnetometer. Additional characterization details are provided in Additional file 1.

### SPION application

MRI experiments were performed on a 3T clinical MR scanner (Philips Achieva, Best, The Netherlands) and images were acquired using SENSE-flex-M coil (Philips Achieva, Best, The Netherlands). From MRI tests, R_1_ and R_2_ relaxation rates and corresponding r_1_ and r_2_ relaxivities were calculated [44]. MPI measurements were performed using the Philips pre-clinical demonstrator system and relevant parameters of the SPION were determined including the signal-to-noise ratio (SNR) and the full width at half maximum (FWHM) of the point spread function (PSF). In order to evaluate hyperthermia performance, a custom-build setup (Trumpf Hüttinger, Freiburg, Germany) was employed and the heating efficiency of the different SPION formulations under an alternating magnetic field (AMF) was quantified using the specific absorption rate (SAR), which provides a measure of the magnetic power absorbed per unit mass of the magnetic material (see Additional file 1 for more details).

## Supplementary information

**Additional file 1: Figure S1.** Zeta potential analysis of the crude, C1-C5, Resovist® and Sinerem® samples. **Figure S2.** Cell viability of NIH3T3 cells treated with the samples with various concentrations ofSPION for 4 h according to XTT assay. The data were normalized to control value (SPION-freemedia), which was set as 100% cell viability. Experiments were performed at different concentrationsof SPION in the range of 0.1 to 10.0 mM. Values represent means ± standard deviations of fiveidentical experiments made in three replicates. **Figure S3.** LDH leakage of NIH3T3 cells treated with the samples with various concentrations ofSPION for 4 h according to the manufacturer’s instructions. Experiments were done at differentconcentrations of SPION in the range of 0.1 to 10.0 mM. Values represent mean ± standard deviationof five identical experiments made in three replicates. **Figure S4.** ROS generated in NIH3T3 cells incubated with the samples with various concentrations ofSPION to the control cells (SPION-free media) after 24 h treatment. Experiments were done atdifferent concentrations of SPION in the range of 0.1 to 5 mM. Data represent mean ± standarddeviation of three identical experiments made in three replicates. **Figure S5.** Colloidal stability of the samples in undiluted FBS monitored by visual inspection andDLS. Visual inspection indicated no aggregation up until 24 h. In line with this, size and PDI obtainedby DLS also showed no significant changes at 24 h. The iron concentration for all the samples was 5mM. The FBS size according to DLS was 19.7±1.5 nm which is very close to hydrodynamic diameterof C1. Also, FBS is polydisperse and has PDI of 0.49±0.05. These two notions explain the high PDI forC1 in FBS. **Figure S6.** Colloidal stability of the samples in 4 wt% BSA in DI water. Visual inspection showed noaggregation at 24 h. Also, size and PDI obtained by DLS showed no important differences in theirvalues at 24 h. The iron concentration for all the samples was 5 mM. **Figure S7.** Temperature-dependent magnetization at 1000 Oe of the crude SPION mixture (C), thesize-isolated samples C1-C5, Resovist® (R) and Sinerem® (S). Results were normalized to Fe content. **Figure S8.** T1- and T2-weighted MR images of the crude, C1-C5, Resovist® and Sinerem® samples atdifferent concentrations from 0.005 to 0.05 mM. **Figure S9.** Longitudinal (1/T1; a) and transverse (1/T2; b) relaxation rates of the crude, C1-C5,Resovist® and Sinerem® samples as a function of concentration of Fe. The straight lines represent thelinear fit to the experimental data. The relaxivities r1 and r2 were calculated as the slope of the linesfitted to the experimental data. Values represent average of one experiment made in three replicates. **Figure S10.** Relaxivity ratios (r_2_/r_1_) for the crude, C1-C5, Resovist® and Sinerem® samples. **Figure S11.** Normalized SNR values of the samples from the 4th up to the 10th harmonic of the MPIdrive field. **Figure S12.** Magnetic particle imaging of size-isolated SPION. (a) MPI images reconstructed basedon “V” shaped phantoms filled with the crude sample, C2 and Resovist®. (b) The intensity line profilesof the red marked lines through the phantoms in panel (a) are shown. The line profiles show the voxelintensity along the marked line and demonstrate a doubling of signal intensity for C2 in comparison toResovist®. **Table S1.** Overview of the results obtained in the size analyses performed using TEM, DLS and NTA.The different SPION formulations were evaluated in different media and upon different storage times.

## Data Availability

Data and materials will be made available upon request.
