# Supplementary material for: Size-isolation of superparamagnetic iron oxide nanoparticles improves MRI, MPI and hyperthermia performance
Source: J Nanobiotechnology. 2020 Jan 28;18:22. doi: 10.1186/s12951-020-0580-1 (PMC6986086; doi:10.1186/s12951-020-0580-1)
Supplement: Supplementary file 1 — Additional file 1: Figure S1. Zeta potential analysis of the crude, C1-C5, Resovist® and Sinerem® samples. Figure S2. Cell viability of NIH3T3 cells treated with the samples with various concentrations ofSPION for 4 h according to XTT assay. The data were normalized to control value (SPION-freemedia), which was set as 100% cell viability. Experiments were performed at different concentrationsof SPION in the range of 0.1 to 10.0 mM. Values represent means ± standard deviations of fiveidentical experiments made in three replicates. Figure S3. LDH leakage of NIH3T3 cells treated with the samples with various concentrations ofSPION for 4 h according to the manufacturer’s instructions. Experiments were done at differentconcentrations of SPION in the range of 0.1 to 10.0 mM. Values represent mean ± standard deviationof five identical experiments made in three replicates. Figure S4. ROS generated in NIH3T3 cells incubated with the samples with various concentrations ofSPION to the control cells (SPION-free media) after 24 h treatment. Experiments were done atdifferent concentrations of SPION in the range of 0.1 to 5 mM. Data represent mean ± standarddeviation of three identical experiments made in three replicates. Figure S5. Colloidal stability of the samples in undiluted FBS monitored by visual inspection andDLS. Visual inspection indicated no aggregation up until 24 h. In line with this, size and PDI obtainedby DLS also showed no significant changes at 24 h. The iron concentration for all the samples was 5mM. The FBS size according to DLS was 19.7±1.5 nm which is very close to hydrodynamic diameterof C1. Also, FBS is polydisperse and has PDI of 0.49±0.05. These two notions explain the high PDI forC1 in FBS. Figure S6. Colloidal stability of the samples in 4 wt% BSA in DI water. Visual inspection showed noaggregation at 24 h. Also, size and PDI obtained by DLS showed no important differences in theirvalues at 24 h. The iron concentration for all the samples was 5 mM. Fig [file 12951_2020_580_MOESM1_ESM.docx]

**- Additional Information -**

**Size-Isolation of Superparamagnetic Iron Oxide Nanoparticles**

**Improves MRI, MPI and Hyperthermia Performance**

Seyed Mohammadali Dadfar,^a^ Denise Camozzi,^b^ Milita Darguzyte,^a^ Karolin Roemhild,^a^

Paola Varvarà,^c^ Josbert Metselaar,^a^ Srinivas Banala,^a^ Marcel Straub,^a^ Nihan Güvener,^a^

Ulrich Engelmann,^d^ Ioana Slabu,^d^ Miriam Buhl,^e^ Jan van Leusen,^f^ Paul Kögerler,^f^

Benita Hermanns-Sachweh,^e^ Volkmar Schulz,^a^ Fabian Kiessling,^a^ Twan Lammers^a,g,h*^

^a^ Institute for Experimental Molecular Imaging, Faculty of Medicine, RWTH Aachen University, Forckenbeckstr. 55, 52074, Aachen, Germany

^b^ Department of Biomolecular Sciences, University of Urbino ‘Carlo Bo’, Via Aurelio Saffi 2, 61029, Urbino, Italy

^c^ Department of Scienze e Tecnologie Biologiche, Chimiche e Farmaceutiche (STEBICEF), University of Palermo, Palermo, Italy

^d^ Institute of Applied Medical Engineering, Helmholtz Institute, Faculty of Medicine, RWTH Aachen University, Pauwelsstrasse 20, 52074 Aachen, Germany

^e^ Electron Microscopy, Institute of Pathology, Faculty of Medicine, RWTH Aachen University, Pauwelstrasse 30, 52074, Aachen, Germany

^f^ Institute of Inorganic Chemistry*,* RWTH Aachen University, Landoltweg 1, 52056 Aachen, Germany

^g^ Department of Pharmaceutics, Utrecht University, Utrecht, The Netherlands

^h^ Department of Targeted Therapeutics, University of Twente, Enschede, The Netherlands

* Corresponding author. Email: tlammers@ukaachen.de

**Additional Figures**

**Figure S1:**

**
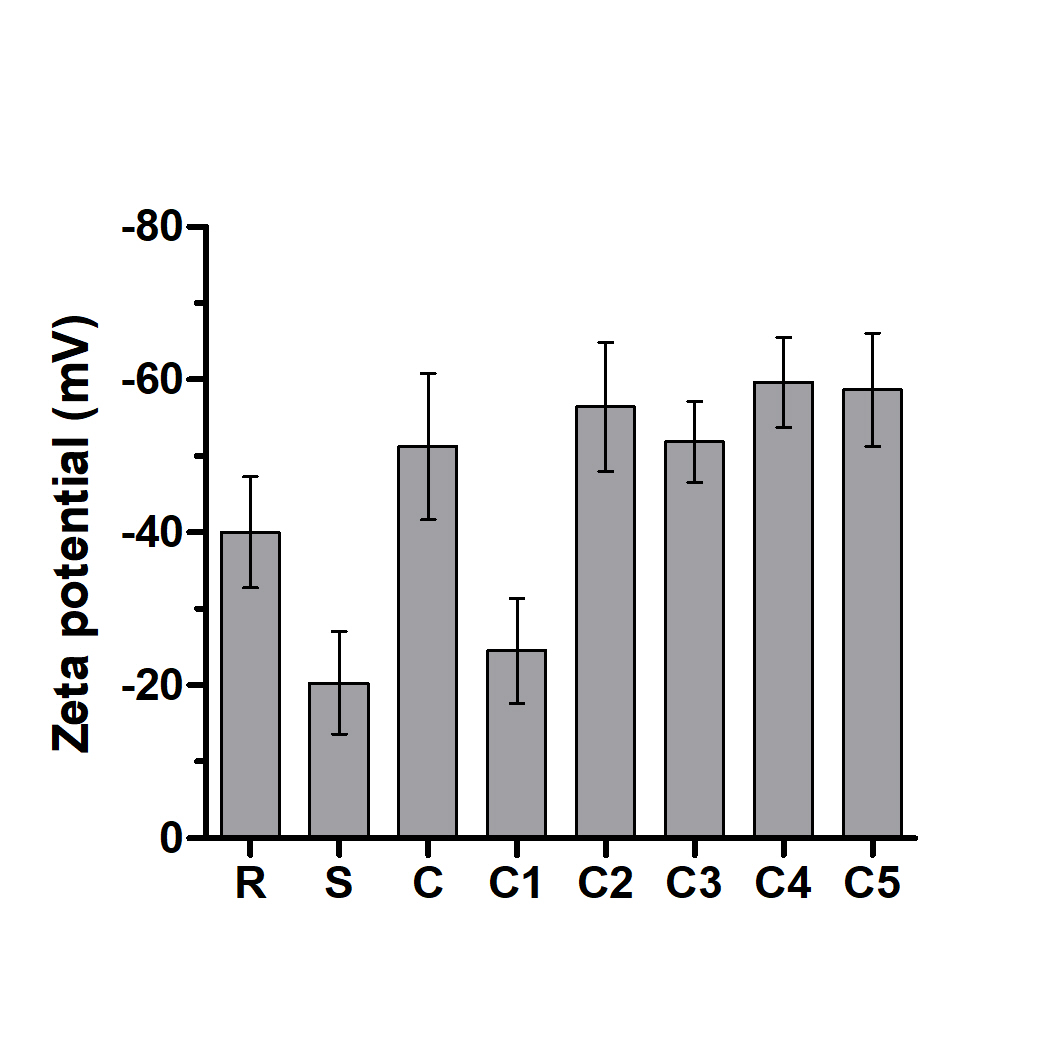
**

**Figure S1.** Zeta potential analysis of the crude, C1-C5, Resovist® and Sinerem® samples.

**Figure S2:**


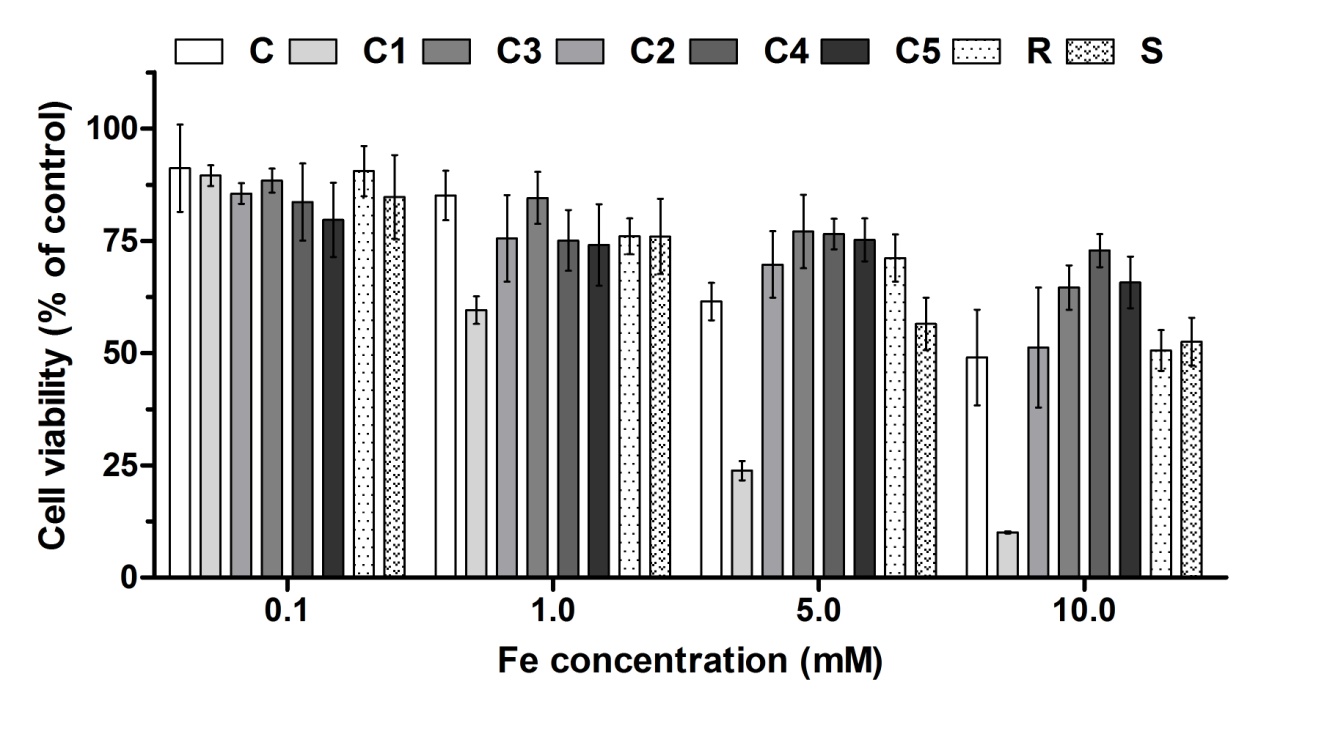


**Figure S2.** Cell viability of NIH3T3 cells treated with the samples with various concentrations of SPION for 4 h according to XTT assay. The data were normalized to control value (SPION-free media), which was set as 100% cell viability. Experiments were performed at different concentrations of SPION in the range of 0.1 to 10.0 mM. Values represent means ± standard deviations of five identical experiments made in three replicates.

**Figure S3:**


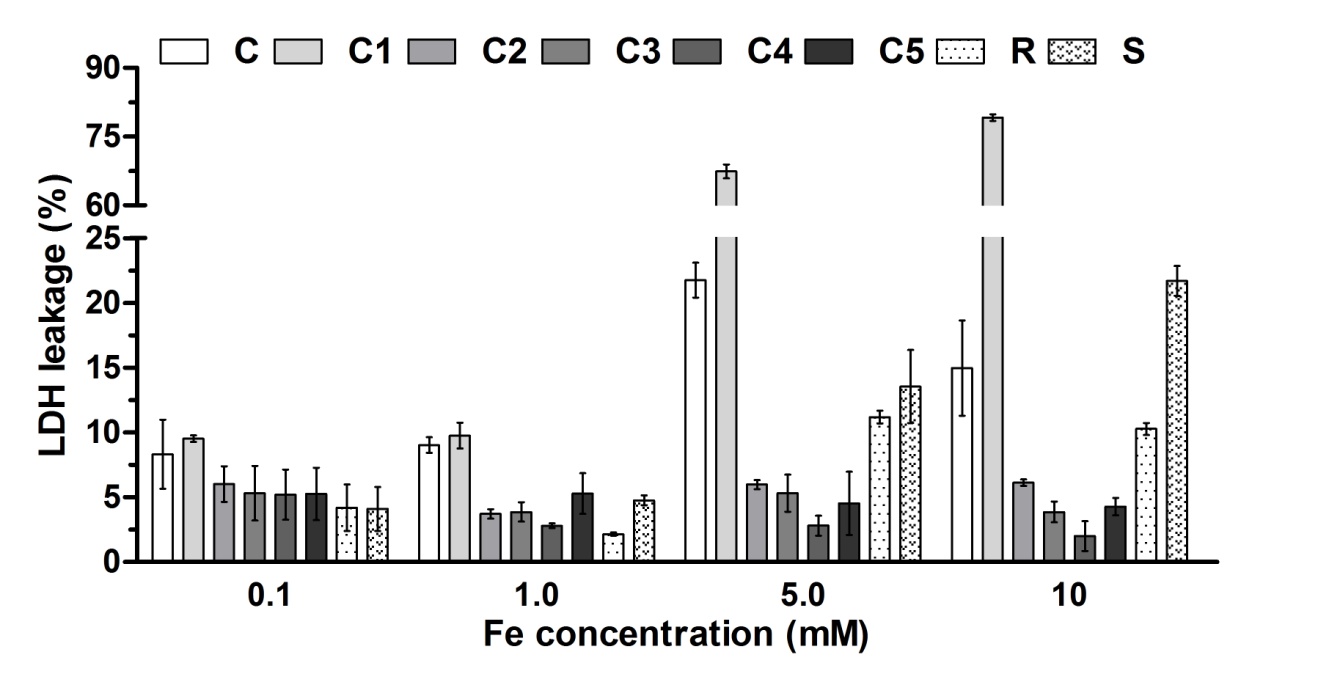


**Figure S3.** LDH leakage of NIH3T3 cells treated with the samples with various concentrations of SPION for 4 h according to the manufacturer’s instructions. Experiments were done at different concentrations of SPION in the range of 0.1 to 10.0 mM. Values represent mean ± standard deviation of five identical experiments made in three replicates.

**Figure S4:**


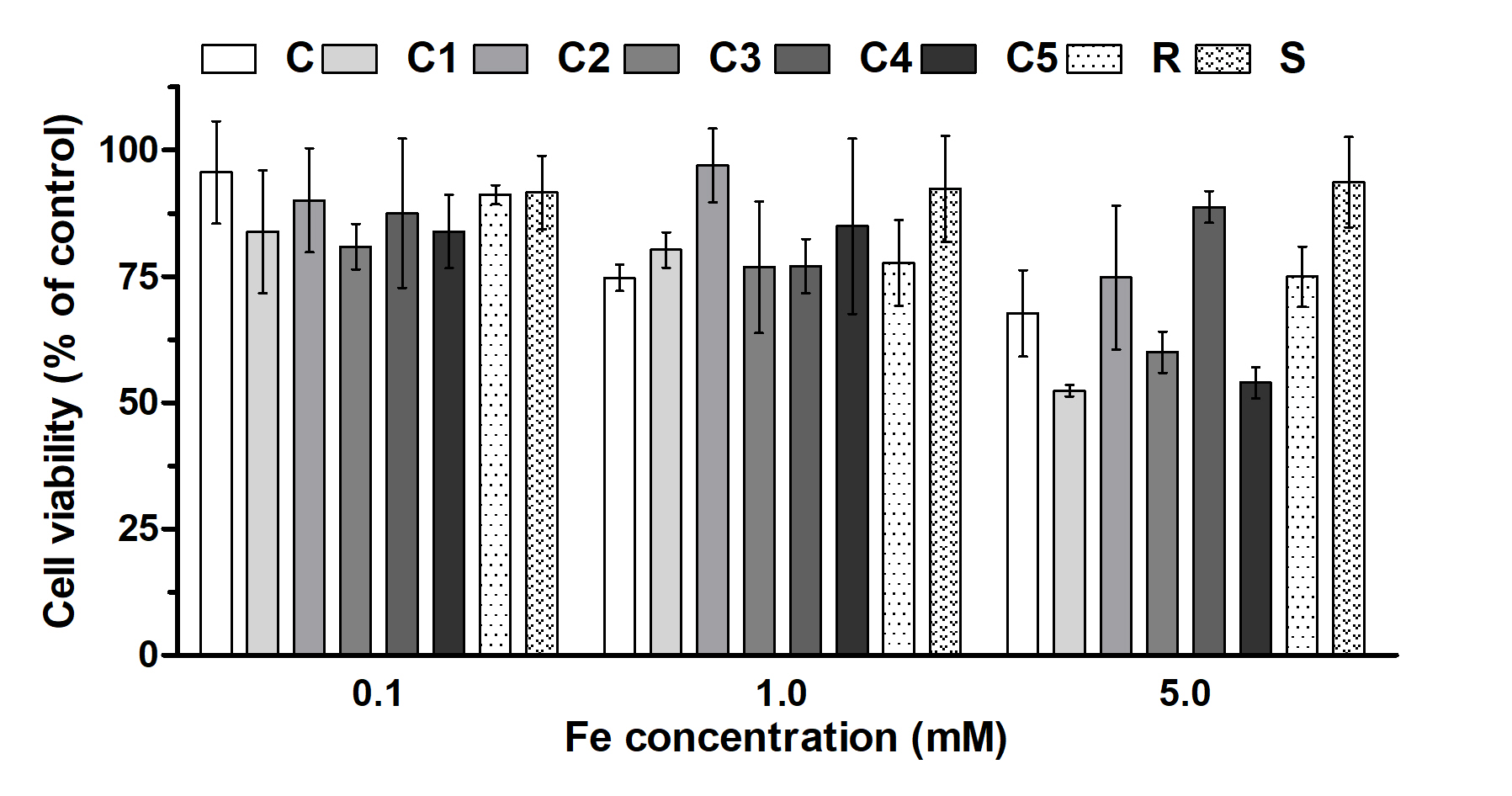


**Figure S4.** ROS generated in NIH3T3 cells incubated with the samples with various concentrations of SPION to the control cells (SPION-free media) after 24 h treatment. Experiments were done at different concentrations of SPION in the range of 0.1 to 5 mM. Data represent mean ± standard deviation of three identical experiments made in three replicates.

**Figure S5:**

**
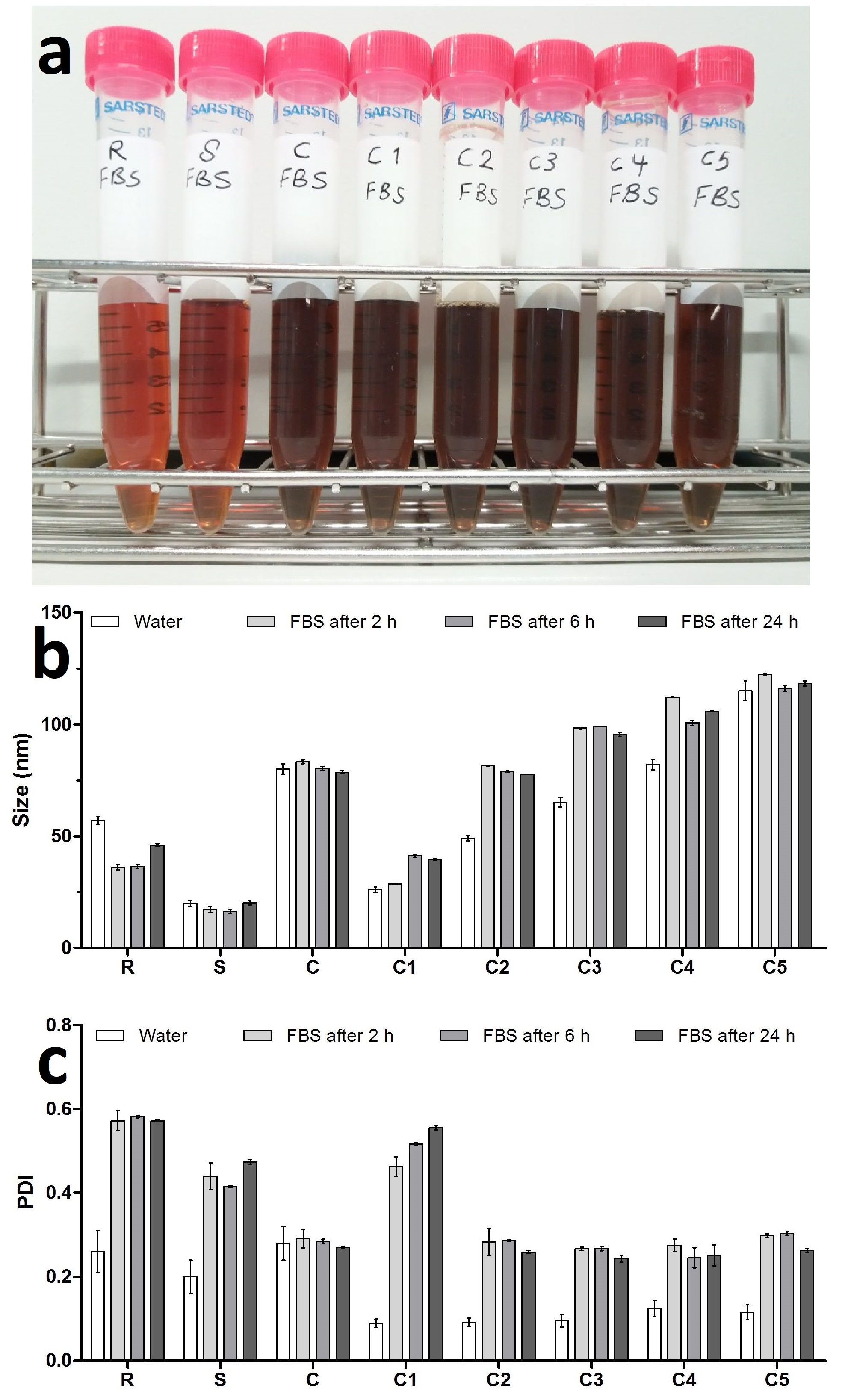
**

**Figure S5.** Colloidal stability of the samples in undiluted FBS monitored by visual inspection and DLS. Visual inspection indicated no aggregation up until 24 h. In line with this, size and PDI obtained by DLS also showed no significant changes at 24 h. The iron concentration for all the samples was 5 mM. The FBS size according to DLS was 19.7±1.5 nm which is very close to hydrodynamic diameter of C1. Also, FBS is polydisperse and has PDI of 0.49±0.05. These two notions explain the high PDI for C1 in FBS.

**Figure S6:**

**
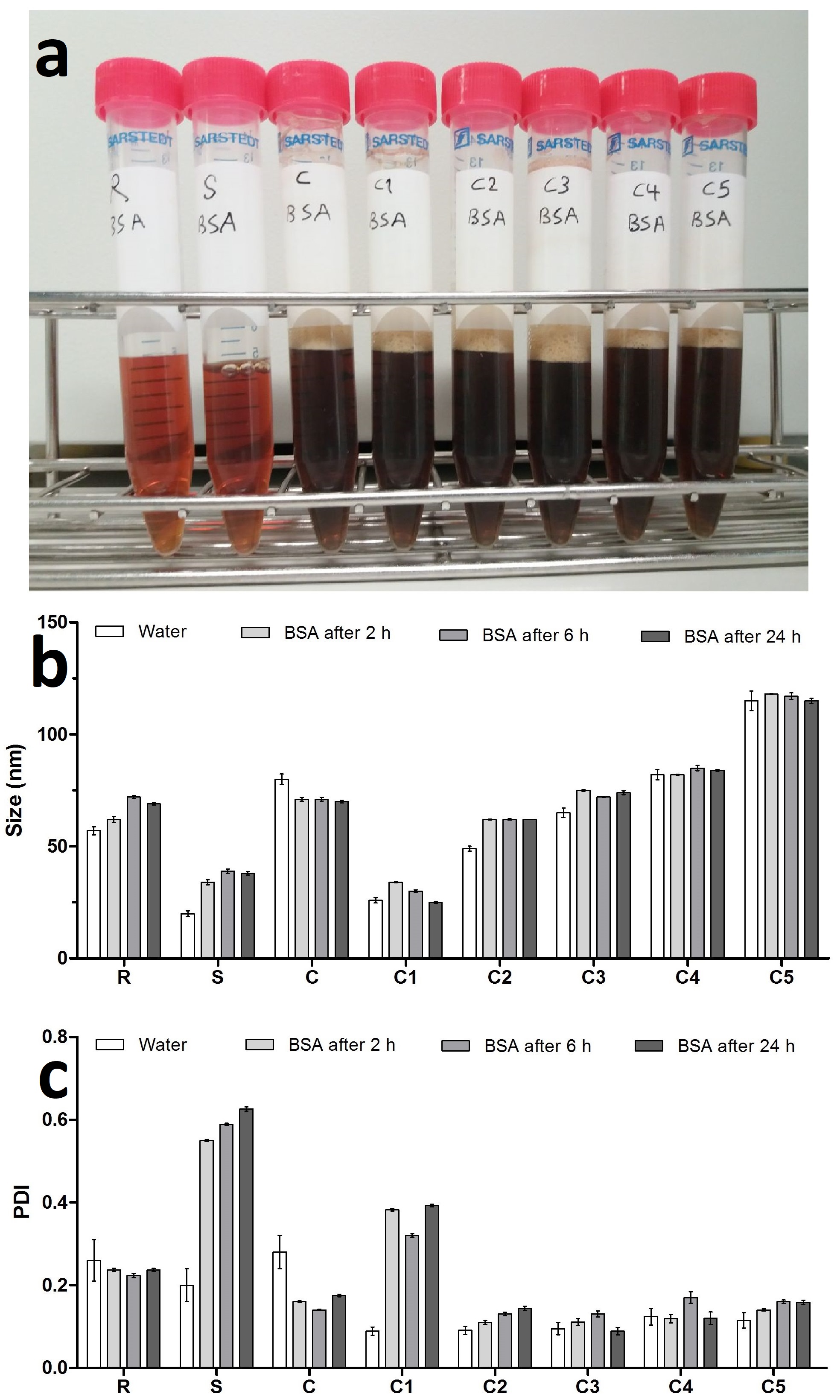
**

**Figure S6.** Colloidal stability of the samples in 4 wt% BSA in DI water. Visual inspection showed no aggregation at 24 h. Also, size and PDI obtained by DLS showed no important differences in their values at 24 h. The iron concentration for all the samples was 5 mM.

**Figure S7:**


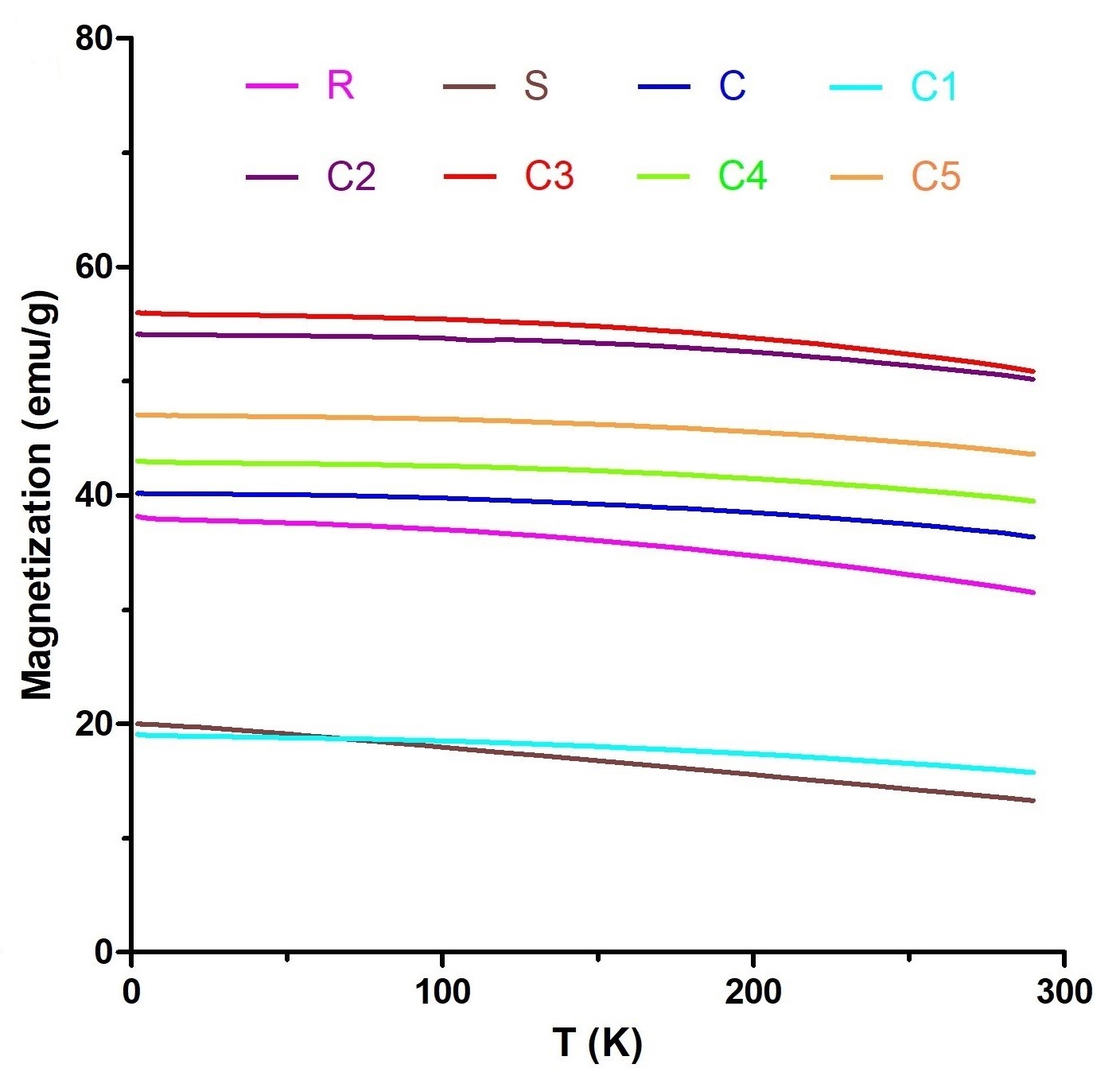


**Figure S7.** Temperature-dependent magnetization at 1000 Oe of the crude SPION mixture (C), the size-isolated samples C1-C5, Resovist® (R) and Sinerem® (S). Results were normalized to Fe content.

**Figure S8:**

| 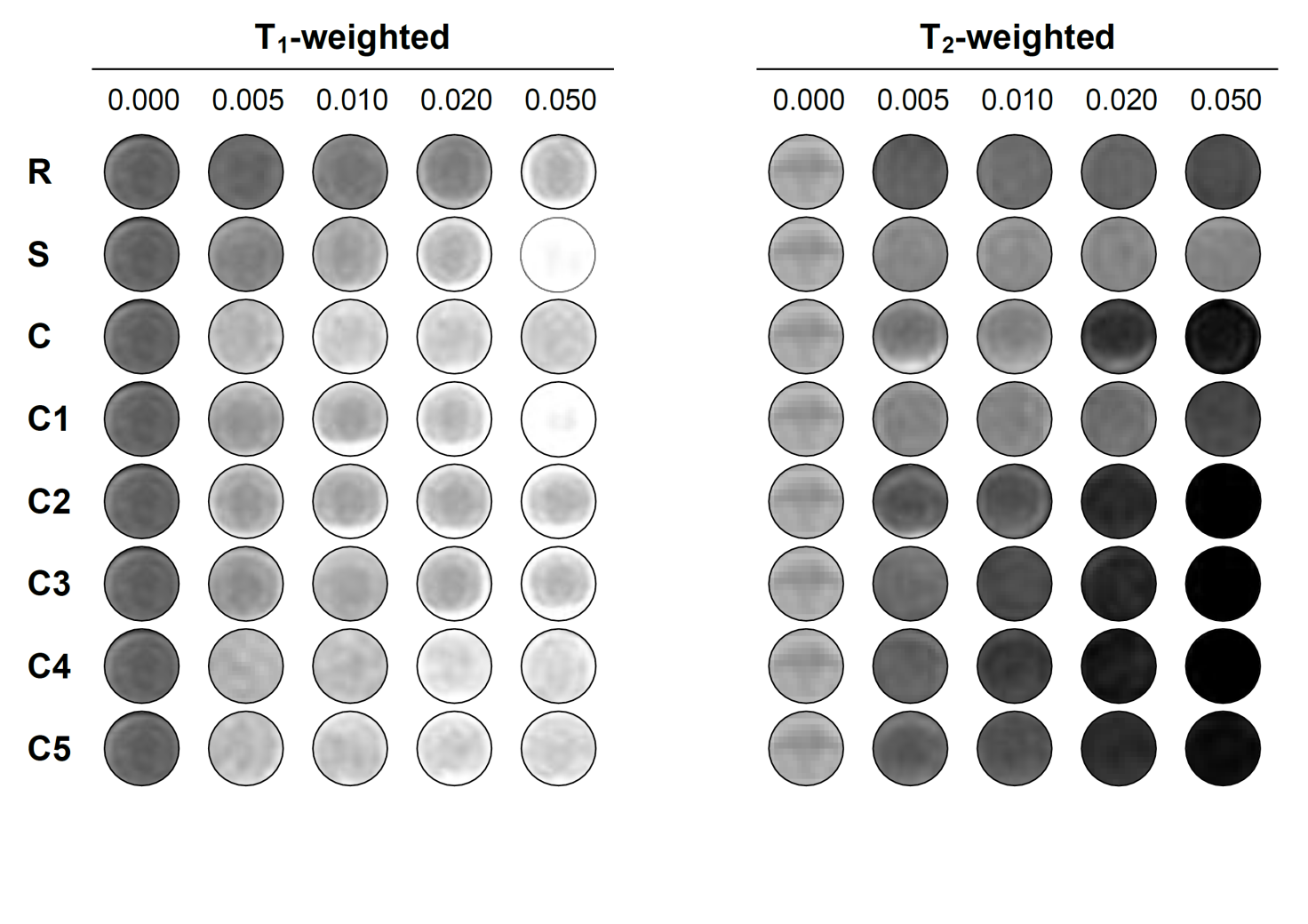 |
| --- |

**Figure S8.** T_1_- and T_2_-weighted MR images of the crude, C1-C5, Resovist® and Sinerem® samples at different concentrations from 0.005 to 0.05 mM.

**Figure S9:**

**
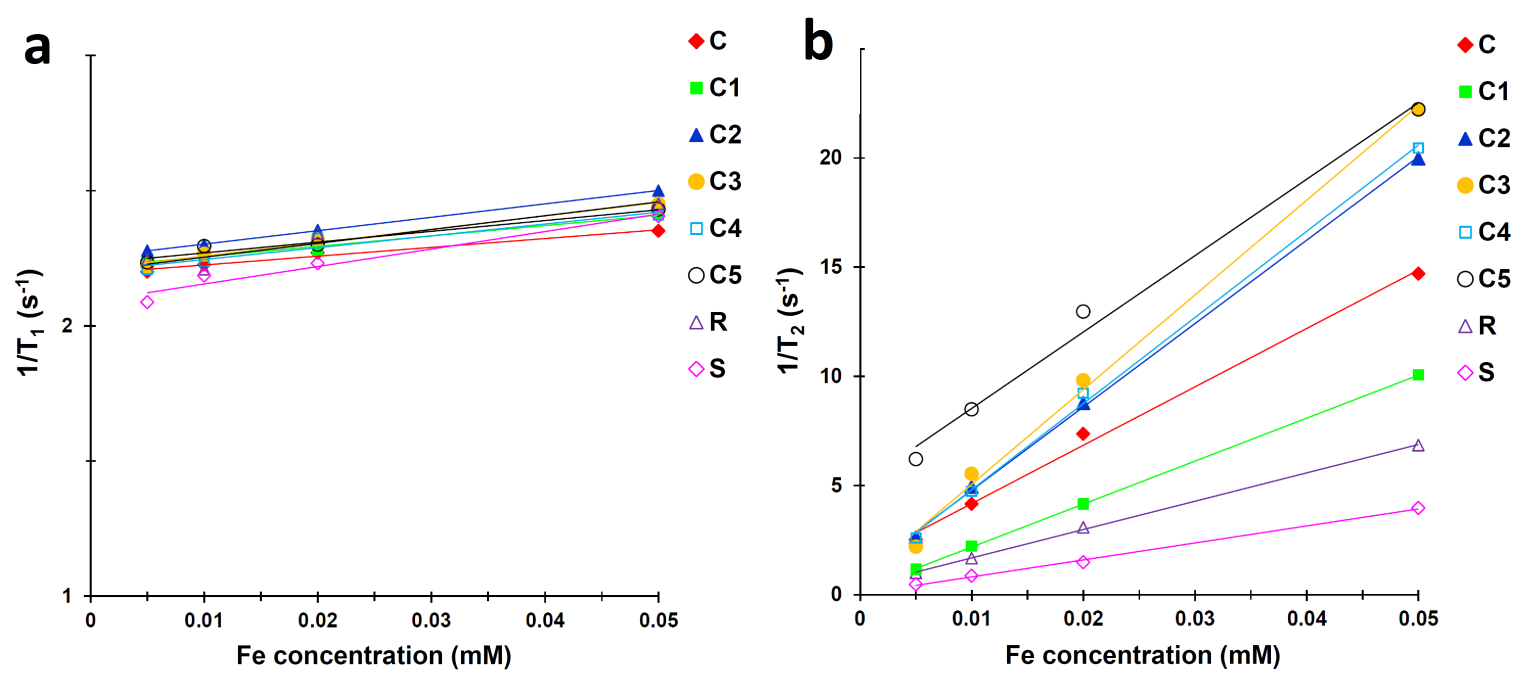
**

**Figure S9.** Longitudinal (1/T_1_; a) and transverse (1/T_2_; b) relaxation rates of the crude, C1-C5, Resovist® and Sinerem® samples as a function of concentration of Fe. The straight lines represent the linear fit to the experimental data. The relaxivities r_1_ and r_2_ were calculated as the slope of the lines fitted to the experimental data. Values represent average of one experiment made in three replicates.

**Figure S10:**


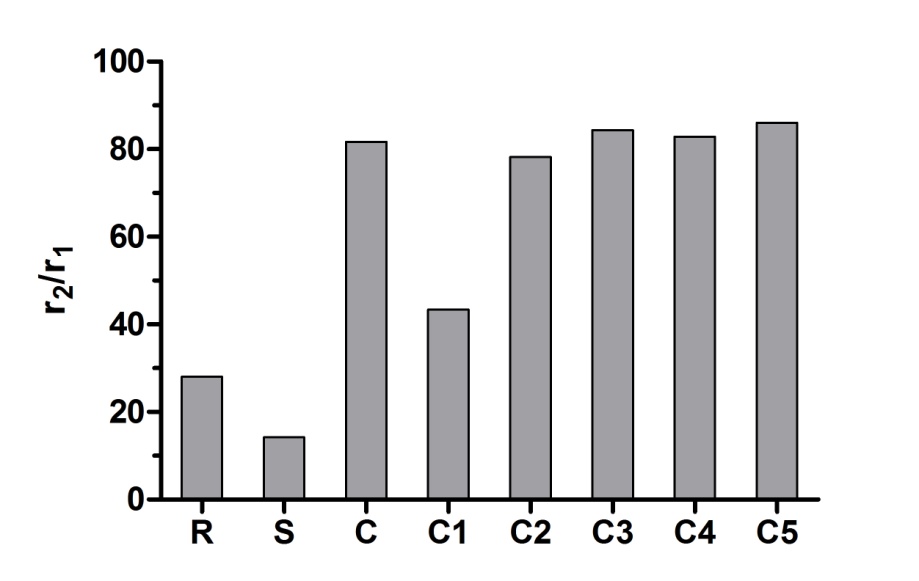


**Figure S10.** Relaxivity ratios (r_2_/r_1_) for the crude, C1-C5, Resovist® and Sinerem® samples.

**Figure S11:**


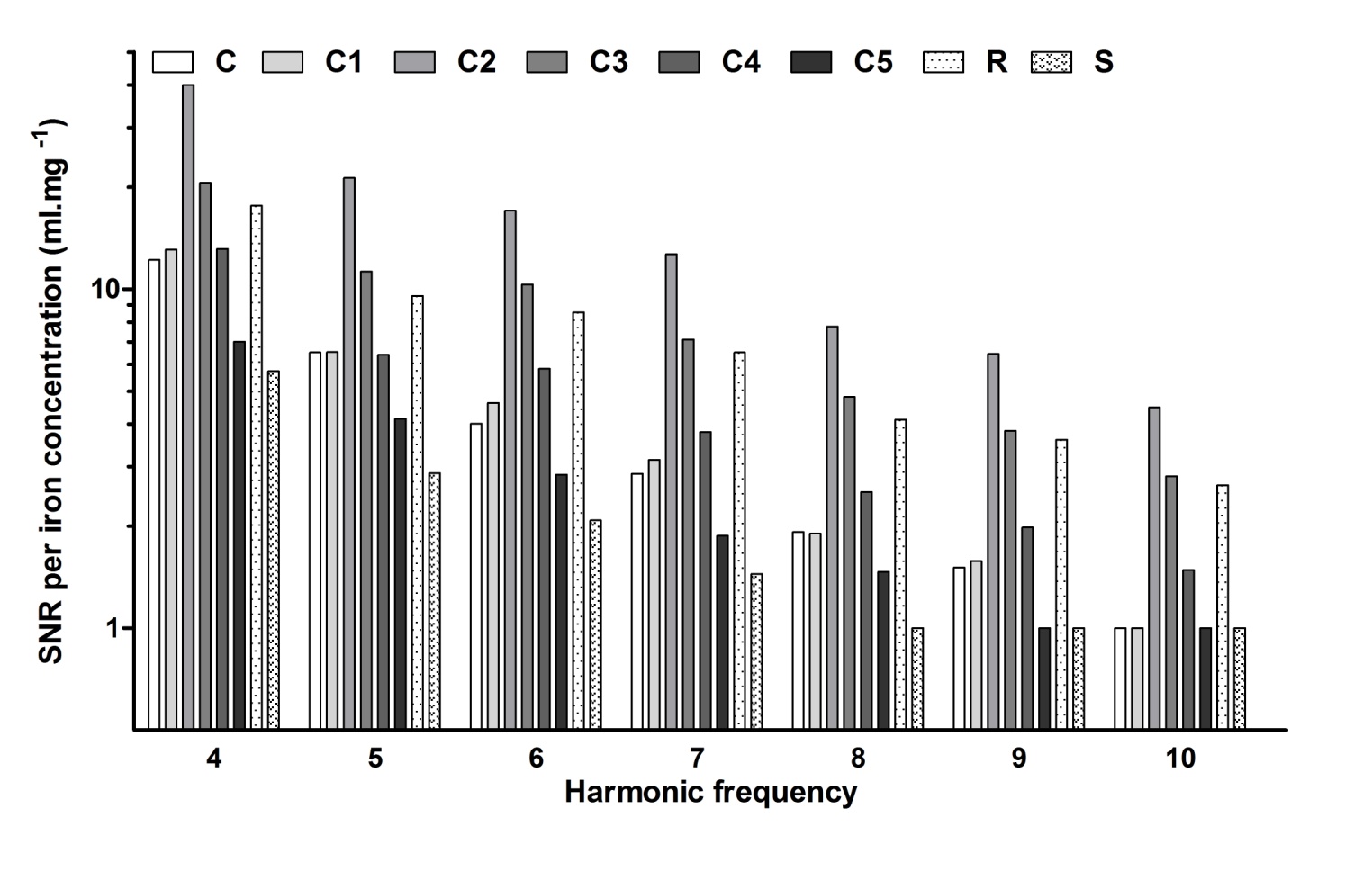


**Figure S11.** Normalized SNR values of the samples from the 4^th^ up to the 10^th^ harmonic of the MPI drive field.

**Figure S12:**


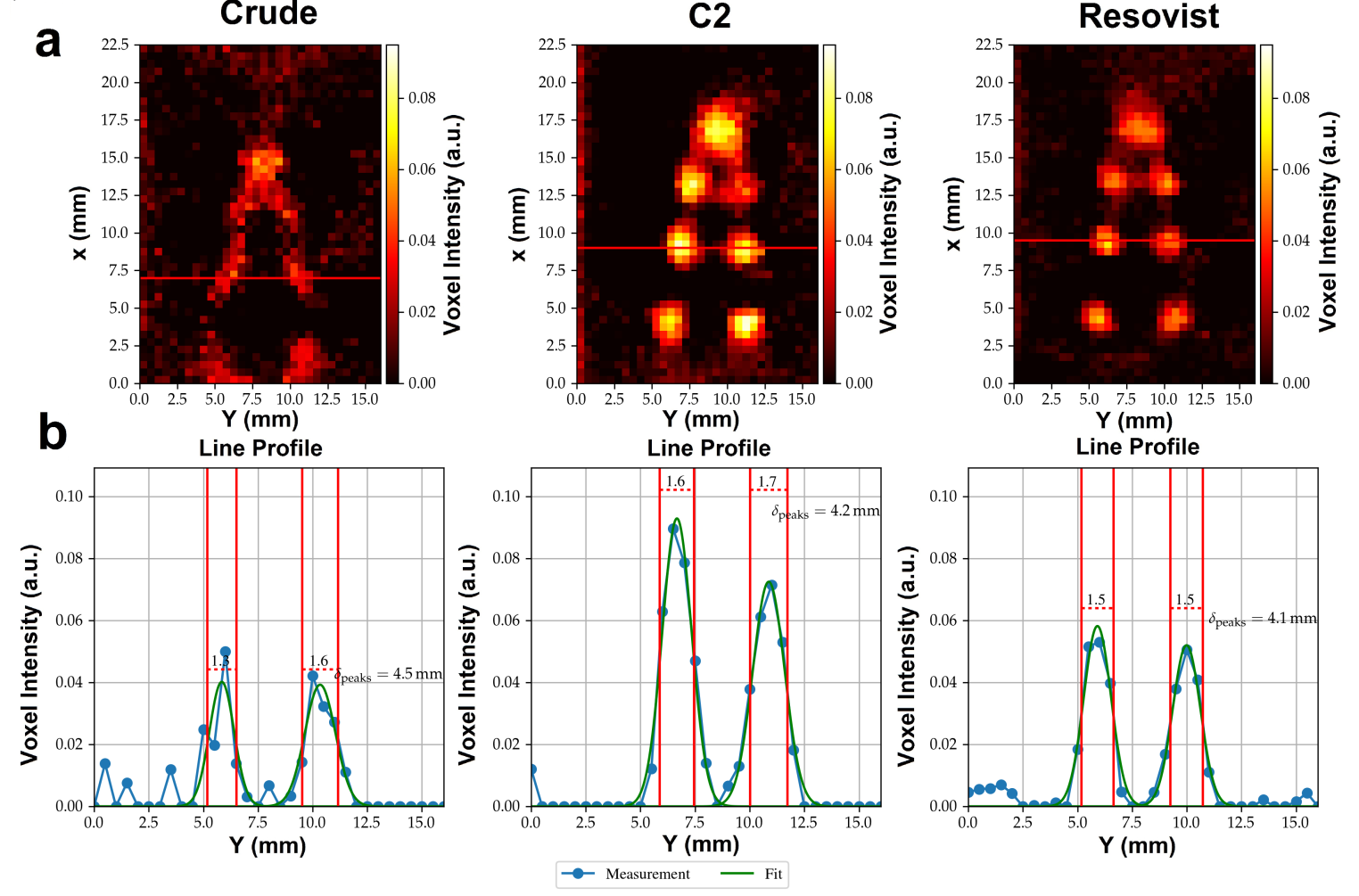


**Figure S12.** **Magnetic particle imaging of size-isolated SPION.** (a) MPI images reconstructed based on “V” shaped phantoms filled with the crude sample, C2 and Resovist®. (b) The intensity line profiles of the red marked lines through the phantoms in panel (a) are shown. The line profiles show the voxel intensity along the marked line and demonstrate a doubling of signal intensity for C2 in comparison to Resovist®.

**Table S1:**

**Table S1.** Overview of the results obtained in the size analyses performed using TEM, DLS and NTA. The different SPION formulations were evaluated in different media and upon different storage times.

| **Sample** | **TEM** | **NTA (SPION in DI water,**  **after 24 hours)** | **DLS (SPION in DI water,**  **after 24 hours)** | **DLS (SPION in DI water,**  **after 6 months)** | **DLS (SPION in FBS,**  **after 24 hours)** | **DLS (SPION in BSA,**  **after 24 hours)** |
| --- | --- | --- | --- | --- | --- | --- |
| R | 5.8 ± 2.5 | 61.0 ± 4.2 | 57.1 ± 1.8 | 59.2 ± 2.3 | 46.1 ± 0.6 | 69.2 ± 0.6 |
| S | 2.5 ± 1.0 | 23.3 ± 4.3 | 20.0 ± 1.3 | 20.5 ± 1.1 | 20.1 ± 0.9 | 38.9 ± 0.8 |
| C | 16.7 ± 7.3 | 90.8 ± 4.4 | 80.4 ± 2.3 | 79.3 ± 2.8 | 78.6 ± 0.7 | 70.7 ± 0.7 |
| C1 | 7.7 ± 1.6 | 30.1 ± 3.1 | 26.3 ± 1.2 | 28.0 ± 0. 9 | 39.6 ± 0.3 | 25.9 ± 0.5 |
| C2 | 10.6 ± 1.8 | 48.4 ± 3.4 | 49.4 ± 1.1 | 51.8 ± 2.6 | 77.6 ± 0.1 | 62.2 ± 0.1 |
| C3 | 13.1 ± 2.2 | 68.1 ± 4.0 | 64.8 ± 2.1 | 65.3 ± 1.0 | 95.4 ± 0.8 | 74.6 ± 0.9 |
| C4 | 15.6 ± 2.8 | 83.4 ± 5.3 | 82.1 ± 2.3 | 84.4 ± 3.1 | 105.8 ± 0.2 | 84.7 ± 0.4 |
| C5 | 17.2 ± 2.1 | 110.5 ± 5.5 | 114.6 ± 4.4 | 119.6 ± 5.8 | 118.2 ± 1.1 | 115.6 ± 1.1 |

**- Additional Materials and Methods -**

**Chemicals**

Ferric chloride anhydrous, ferrous chloride tetrahydrate, trisodium citrate dihydrate, 1,10-phenanthroline, hydroxylamine hydrochloride and ammonium iron (II) sulfate hexahydrate were purchased from Sigma-Aldrich (Munich, Germany). Hydrogen chloride, potassium hydroxide and ammonium hydroxide were obtained from Carl Roth (Karlsruhe, Germany). Resovist® (Bayer Schering Pharma AG, Berlin, Germany) and Sinerem® (Guerbet, France) were used as commercial SPION brands. Colorless DMEM media, phosphate-buffered saline (PBS), fetal bovine serum (FBS), penicillin/streptomycin and trypsin were bought from Invitrogen (AG, Basel, Switzerland). Nitroblue-tetrazoliumchloride (NBT) was ordered from AppliChem (Darmstadt, Germany).

**Dynamic light scattering (DLS)**

The zeta potential, average hydrodynamic diameter (D_h_) and polydispersity index (PDI) of the particles were measured using a Zetasizer Nano-ZS instrument (Malvern Instruments, Malvern, UK) at 25 °C. The DLS machine was equipped with a 633 nm He–Ne laser and a detector at angle of 173°. The samples were diluted with DI water and sonicated in a water bath prior to size analysis. To investigate the stability of the particles, the experiments were repeated every two weeks. Each sample was analyzed three times.

**Transmission electron microscopy (TEM)**

For the TEM measurements, the samples were diluted in DI water and sonicated in order to reduce agglomeration. The diluted dispersions were then dropped onto carbon-coated copper grids and dried with filter papers located underneath at room temperature. TEM images were obtained using a Zeiss 906 electron microscope (LEO, Oberkochen, Germany) operated at 60 kV. From the TEM image and using the software Image SP Viewer software, the size of at least 100 particles was determined and then the mean particles sizes were calculated manually.

**Nanoparticle tracking analysis (NTA)**

NTA measurements were performed using a NanoSight NS300 system (NanoSight Ltd., Malvern, UK).The samples were diluted with DI water to the suitable concentration for the analysis. The diluted samples were injected into the viewing chamber equipped with a 532 nm laser at 25 °C. Videos tracking of the particles were recorded with a CCD camera (Allied Vision Technologies, Germany) and NanoSight 2.2 software was used for capturing and analyzing the samples data.

**Iron quantification**

One milliliter of the diluted SPION was taken and ionized by adding 0.5 ml of the concentrated HCl. The solution was heated until the color changed to yellow. 1.0 ml hydroxylamine hydrochloride solution was added to reduce ferric ion to ferrous form. The sample was stirred and then 1.0 ml of the 1,10-phenanthroline solution was added to form an orange-red complex with ferrous ions. The mixture was neutralized with sodium hydroxide and the pH of the solution was adjusted to about 4.0 by adding citrate buffer. The solution was mixed well and kept for 10 min in order to color development. Finally, the absorbance was detected at 510 nm using an Infinite M200 Pro TECAN reader (TECAN, Germany). In addition, a standard calibration curve was made with several amounts of ammonium iron (II) sulfate hexahydrate.

**Magnetic properties**

The magnetic properties of samples, including field-dependent magnetization, saturation magnetization (M_s_) and field-cooled (FC) magnetization, were determined using a magnetometer. The freeze-dried powder samples were compacted and immobilized into cylindrical PTFE capsules. Field-dependent magnetization and saturation magnetization were acquired as a function of magnetic field at 5.0 K. FC magnetization measurements were carried out in an applied field of 1000 Oe from 5 to 300 K. Data were corrected for the diamagnetic contributions of sample holders and compounds. The obtained parameters were normalized to Fe content.

**Magnetic resonance imaging (MRI)**

The MRI samples were prepared at concentrations ranging from 0.005 to 0.05 mM Fe. For the MR measurements, all the samples were placed on 96 well plates as triplicates of 0.2 ml/well. As control, Resovist® and Sinerem® were used as commercially available SPION and water was used to set the background in the absence of the SPION particles. T_1_ and T_2_ relaxation times and corresponding R_1_ and R_2_ relaxation rates were calculated by fitting an exponential curve to the signal amplitudes using the Imalytics Preclinical Software [1]. MRI experiments were performed on a 3T clinical MR scanner (PHILIPS Achieva, The Netherlands) and images were acquired using SENSE-flex-M coil (PHILIPS, The Netherlands). T2-weighted images were acquired in multi-slice, turbo spin echo (TSE) sequence [repetition time (TR) = 846 ms, echo time (TE) = 100 ms, flip angle (FA) = 90°, slice thickness = 2 mm and field of view (FOV) = 180 mm] and T1-weighted images were acquired in TSE sequence [TR = 500 ms, TE = 15 ms, FA = 75°, slice thickness = 5 mm and FOV = 150 mm].

T2-weighted relaxometry data were obtained from multi-slice (MS), TSE sequence [TR = 1500 ms, TE = 8-162 ms, FA = 90°, slice thickness = 3 mm and FOV = 130 mm] and T1-weighted relaxometry data were obtained from 2D scan mode turbo field echo (TFE) sequence [TR = 7 ms, TE = 3 ms, FA = 10°, slice thickness = 5 mm and FOV = 170 mm]. The sample concentrations, which refer to the concentration of iron, are obtained via phenanthroline assay. The following equations were fitted to the experimental relaxation data:

| $\frac{1}{T_{1}}=\frac{1}{T_{1}^{0}}+r_{1}.c_{Fe}$ | (S1) |
| --- | --- |
| $\frac{1}{T_{2}}=\frac{1}{T_{2}^{0}}+r_{2}.c_{Fe}$ | (S2) |

where *c_Fe_* stands for the iron concentration and r_1_ and r_2_ are the longitudinal and transverse relaxivity, respectively.

**Magnetic particle imaging (MPI)**

MPI measurements were performed using the Philips pre-clinical demonstrator system. The static selection field had a gradient of 5.5 T/m/µ_0_ in the steepest direction. The three orthogonal dynamic drive fields had an amplitude of 18 mT/µ_0_ and were operated at frequencies of *f_x_* = 26.04 kHz, *f_y_* = 24.51 kHz and *f_z_* = 25.25 kHz. For all spectroscopic MPI measurements, an excitation field strength of 20 mT/µ_0_ at a frequency of 25 kHz was used.

The ability of SPION to provide promising imaging modality in MPI can be expressed by the SNR parameter which is defined according to the following equation:

| $SNR=\frac{P_{\mathrm{signal}}}{P_{\mathrm{noise}}}$ | (S3) |
| --- | --- |

where *P*_signal_ and *P*_noise_ are the power of the sample signal and background [noise](https://en.wikipedia.org/wiki/Noise_(electronic)), respectively. Since different MPI tracers differ in their iron concentration, it is common practice to normalize the SNR measured for a tracer by its iron concentration. For the SNR measurements, a probe head with 0.8 μl of the sample material was positioned into the isocenter of the MPI device.

The PSF of the SPION is the change of magnetization as a function of the applied drive field and is determined from spectroscopic MPI measurements by the method published by Schmale et al. [2]. Based on the FWHM of the PSF, the spatial resolution (Δx) which is achievable in an MPI device with a gradient steepness (G) is given by:

| $\Delta x=G.FWHM$ | (S4) |
| --- | --- |

The latter dependent formulation implicates that the spatial resolution depends linearly on the FWHM. For the spectroscopic PSF measurement, 20 μl of the sample was positioned in the isocenter of the scanner.

**Magnetic fluid hyperthermia (MFH)**

Four milliliters of each sample were placed in glass vials (Rotilabo, Carl Roth GmbH, Karlsruhe, Germany) and inserted in the center of a copper coil (20 mm inner diameter, 30 mm outer diameter, 8 hollow turns) which generated an alternative magnetic field (AMF) with a frequency of 186 ± 3 kHz and an amplitude of 46 ± 2 kA/m (Trumpf Hüttinger, Freiburg, Germany). Using an external cooling unit (Chilly 5 S, Hyfra GmbH, Germany) the interior of the coil turns were flushed with water so that the ambient temperature inside the coil could be stabilized at 37 °C. Increase in the sample temperature as a function of time was recorded using a Luxtron 812 fiber-optic thermometer (LumaSense Inc., Santa Barbara, California, USA). The Fe concentration of all the samples was about 9 mM. Before applying the AMF, all samples were heated up on a hot plate to the physiological temperature of 37 °C which was also set inside the coil. The SAR value, expressed as W/g Fe, is defined according to the following expression [3]:

| $SAR=\frac{C_{w}}{\omega_{\mathrm{Fe}}}\cdot\left. \frac{dT}{dt} \right\vert_{t=0}$ | (S5) |
| --- | --- |

where *C_w_* is the specific heat capacity of water and *ω_Fe_* is the weight fraction of Fe in the sample. The initial slope $\left. \frac{dT}{dt} \right|_{t=0}$is derived from the parameters of Box-Lucas function fit to the temperature vs. time data using the following equations:

| $T\left( t \right)=\Delta T_{rise}.\left( 1-\exp\left( -b .t \right) \right)+T_{0}$ | (S6) |
| --- | --- |
| $\left. \frac{dT}{dt} \right\vert_{t=0}=b.\Delta T_{rise}$ | (S7) |

In equations S6 and S7, b is the time constant of heating obtained from the data fitting. Furthermore, t is time and ΔT_rise_ is the difference between the final temperature (T_max_) and initial temperature (T_0_) in hyperthermia measurements.

Equation S1 can be simplified to:

| $SAR=\frac{C_{w}.\rho_{w}}{c_{Fe}}\cdot\left. \frac{dT}{dt} \right\vert_{t=0}$ | (S8) |
| --- | --- |

where *c_Fe_* and *ρ_w_* are the iron concentration and the water density, respectively. By inserting *C_w_*= 4.18 J/(g.°C), *ρ_w_*= 1 g/ml and *c_Fe_* = 9 mM (0.5 mg/ml), the final form of the SAR equation will be:

| $SAR=8.36\cdot\left. \frac{dT}{dt} \right\vert_{t=0}$ | (S9) |
| --- | --- |

**XTT assay**

Cytotoxicity of the particles was analyzed by monitoring the mitochondrial reduction of XTT assay ordered from American Type Culture Collection (ATCC, Manassas, VA, USA) according to the manufacturer’s instructions. Briefly, NIH3T3 cells (3×10^4^ cells/ml) were seeded in a 96-well plate and incubated overnight. Subsequently, the cells were treated with SPION for 4 h. Then, 50 µl of XTT test solution, which was prepared by mixing 100 μl of the activation reagent with 5 ml of the XTT reagent, was added to each well. After 2 h of incubation, absorbance was measured using a Tecan Infinite M200 plate reader at wavelength of 460 nm.

**LDH assay**

Leakage of lactate dehydrogenase (LDH) was determined using LDH detection kit (Roche Diagnostics GmbH, Mannheim, Germany) according to the manufacturer’s instructions. Briefly, NIH3T3 cells (3×104 cells/ml) were seeded in a 96-well plate and incubated with different concentrations of SPION. After four hours, the supernatant were centrifuged and 100 μl of it was transferred to a new 96-well plate. Finally, 100 μl of the LDH substrate mixture was added to each well and the plates were kept for 30 minutes in the dark at room temperature. The intensity of red color formed in the assay was measured at wavelength of 490 nm by a Tecan Infinite M200 plate reader.

**ROS assay**

Reactive oxygen species (ROS) were quantitated using nitroblue tetrazolium salt (NBT). NIH3T3 cells (3×10^4^ cells/ml) were seeded in a 48-well plate and after overnight incubation with the SPION, media were removed. The cells were washed thrice with PBS and fresh media containing NBT (5 mg/ml) were added and incubated overnight. The supernatant were taken out and the cells were washed with PBS to make sure nothing of the NBT is left in the wells. Then, the trypsin solutions were added to each well and the plates were shaken at 750 rpm for 20 min at ambient temperature. After adding KOH solution, the plates were again shaken at 750 rpm for 30 min. Finally, the absorbance was measured at 620 nm by a Tecan Infinite M200 plate reader.

**References:**

[1] F. Gremse, M. Stärk, J. Ehling, J. R. Menzel, T. Lammers, F. Kiessling, Theranostics 2016, 6, 328.

[2] I. Schmale, J. Rhamer, B. Gleich, J. Borgert, J. Weizenecker, T. Buzug, Magnetic particle imaging, Springer proceedings in physics 2012, 140, 287.

[3] R. Wildeboer, P. Southern, Q. Pankhurst, J. Phys. D: Appl. Phys. 2014, 47, 495003.
